# Synthesis, Bioactivity and Molecular Docking of Nereistoxin Derivatives Containing Phosphonate

**DOI:** 10.3390/molecules28124846

**Published:** 2023-06-19

**Authors:** Qiaoli Yan, Xiaogang Lu, Zixuan Zhang, Qian Jin, Runli Gao, Liqin Li, Hongmei Wang

**Affiliations:** State Key Laboratory of NBC Protection for Civilian, Beijing 102205, China; 17801202159@163.com (Q.Y.); luxg2018@sina.com (X.L.); zzxuan99599@163.com (Z.Z.); jinqian160@163.com (Q.J.); gaorunli@163.com (R.G.)

**Keywords:** nereistoxin, phosphonate, acetylcholinesterase inhibition, insecticidal activity, docking

## Abstract

Novel nereistoxin derivatives containing phosphonate were synthesized and characterized via ^31^P, ^1^H and ^13^C NMR and HRMS. The anticholinesterase activity of the synthesized compounds was evaluated on human acetylcholinesterase (AChE) using the in vitro Ellman method. Most of the compounds exhibited good inhibition of acetylcholinesterase. All of these compounds were selected to assess their insecticidal activity (in vivo) against *Mythimna separata* Walker, *Myzus persicae* Sulzer and *Rhopalosiphum padi*. Most of the tested compounds displayed potent insecticidal activity against these three species. Compound **7f** displayed good activity against all three insect species, showing LC_50_ values of 136.86 μg/mL for *M. separata*, 138.37 μg/mL for *M. persicae* and 131.64 μg/mL for *R. padi*. Compound **7b** had the highest activity against *M. persicae* and *R. padi*, with LC_50_ values of 42.93 μg/mL and 58.19 μg/mL, respectively. Docking studies were performed to speculate the possible binding sites of the compounds and explain the reasons for the activity of the compounds. The results showed that the compounds had lower binding energies with AChE than with the acetylcholine receptor (AchR), suggesting that compounds are more easily bound with AChE.

## 1. Introduction

*Lepidoptera* and *Hemiptera* are destructive pests of many crops in the world. The larvae of many lepidopteran species feed on leaves, and can eat all the leaves of crops when they occur in large areas, causing serious losses of crops [1,2]. Hemipteran pests can also damage crops by causing abnormal plant growth, mold growth and the transmission of plant viruses [2,3,4]. For example, the oriental armyworm (*Mythimna separate* Walker), a typical lepidopteran pest, and aphids, the most difficult hemipteran pests to control in many crops, are widely distributed in areas where rice, wheat and corn are grown. Although various insecticides have been discovered and registered to control lepidopteran and hemipteran pests, the excessive and repeated application of these agricultural chemicals has led to the development of resistance in lepidopteran and hemipteran pest populations and an increase in the burden they place on the environment [5,6].

**Figure 1:**
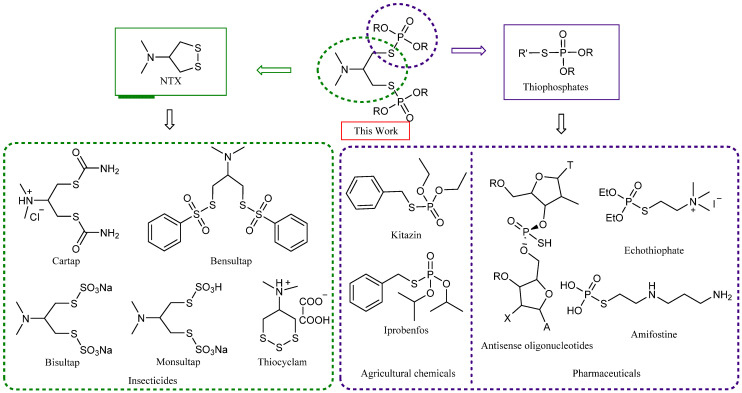
The design of target compounds.

Natural products are the components or secondary metabolites of plants, animals or micro-organisms in nature, which ordinarily are harmless to the environment. An important field of new pesticide research involves discovering and developing new compounds with insecticidal activity from nature or using them as lead compounds for structural modification [7]. Nereistoxin (NTX, Figure 1), a natural small-molecule toxin with insecticidal activity, was isolated from *Lumbriconereis heteropoda*, which lives in marine sediments [8]. It was the first example of an animal toxin that was successfully used as a basis for analog synthesis. Nereistoxin insecticides are effective for lepidopteran, hemipteran and coleopteran pest control. Konishi synthesized and studied the insecticidal activity of many nereistoxin derivatives and established the structure-activity relationship (SAR) of nereistoxin derivatives [9,10,11,12]. However, there are only five insecticide products (Figure 1) currently on the market that use nereistoxin as their active ingredient. The long-term and excessive use of these insecticides will inevitably cause pest resistance. It is necessary to develop an effective way to synthesize new nereistoxin insecticides to extend the application of such insecticides.

Organophosphorus (OP) compounds are an important chemical class with many applications. OPs are used in agricultural chemicals, in medicines, and in the chemical industry and as nerve agents [13,14,15]. Phosphorothioates and dithioates are important classes of OPs that have applications in medicines and pesticides due to their biological properties [16,17,18]. An important characteristic of thiophosphate compounds is that they have low environmental stability in plants, animals and soil micro-organisms and can be rapidly decomposed into less toxic metabolites. This makes them safer for humans, domestic animals and the environment [19].

Zhang et al. reported the synthesis of four phosphorothioate and dithioate nereistoxin derivatives [20,21,22,23]. Their report shows that the dithiotic compounds have excellent insecticidal activity against *Plutella xylostella (Linnaeus)* and wheat aphid. In this work, we developed a series of phosphorothioate nereistoxin derivatives with disulfide bonds opened and modified by phosphate esters in a simple, mild and fast way. The derivatives were characterized via ^1^H, ^13^C and ^31^P nuclear magnetic resonance (NMR) and high-resolution mass spectrometry (HRMS). We studied the insecticidal activity of the derivatives against pre-third-instar larvae of the oriental armyworm (*M. separata*) and aphids (*M. persicae*, *R. padi*) in vivo, as well as their inhibition properties on acetylcholinesterase (AChE) in vitro. Molecular docking was explored to identify the binding site and the reasons for the different potencies of the compounds.

## 2. Results and Discussion

### 2.1. Chemistry

**Scheme 1:**
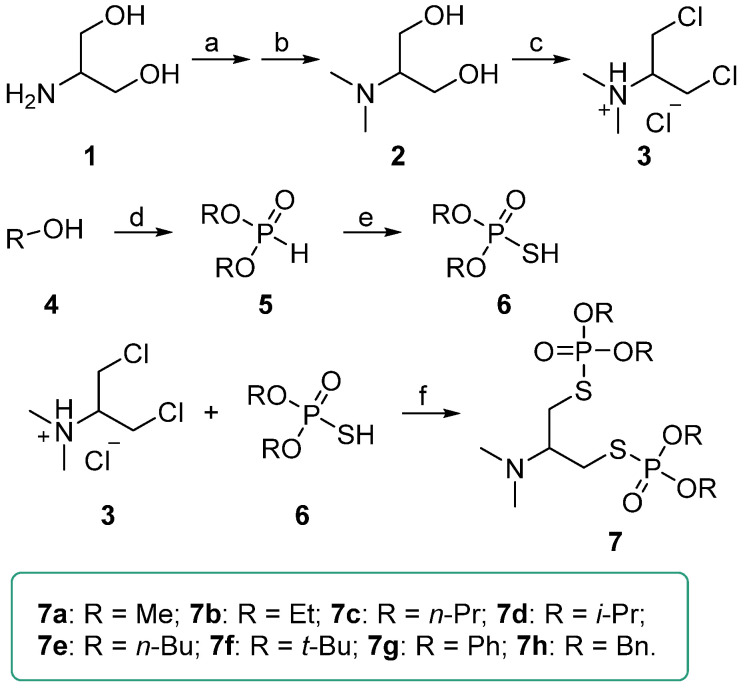
The synthesis process of target compounds **7a**–**7h**. Reagents and conditions: (**a**) HCOOH, CH_2_O (aq., 37%), 60 °C, 3 h; (**b**) 90 °C, 6 h, 84% for **2**; (**c**) SOCl_2_, CHCl_3_, reflux, 4 h, 70% for **3**; (**d**) pyridine, PCl_3_, Et_2_O, 0 °C, 16 h, 51–79% for **5**; (**e**) S_8_, Et_3_N, Et_2_O, *rt*., 12 h, 51–98% for **6**; (**f**) CH_3_CN, NaH, 50 °C, 0.5–5 h, 20–78% for **7**.

The general synthetic route of compounds **7a***–***7h** is shown in Scheme 1. The key intermediate **3** was conveniently synthesized in two steps from 2-amino-1,3-propanediol **1**. The amino group of **1** was dimethylated via the Eschweiler–Clarke reaction, and then, hydroxyl was chlorinated with SOCl_2_ to obtain 1,3-dichloro-*N*,*N*-dimethyl-2-propanamine hydrochloride **3**. Other crucial intermediates, such as *S*-hydrogen phosphorothioates **6**, were obtained from diphosphite **5**. Some of them were commercially available and others were obtained via reaction of the appropriate alcohol and PCl_3_, with pyridine as an acid-binding agent. Diphosphite **5** was reacted with S_8_ and triethylamine to obtain **6**. The end-products of **7** were synthesized via a substitution reaction between intermediates **3** and various substituted *S*-hydrogen phosphorothioates **6**. The target compounds **7a**–**7h** were characterized via ^1^H NMR, ^13^C NMR, ^31^P NMR and HRMS.

### 2.2. Biological Activity

In this study, in accordance with the guidelines for antipesticide laboratory biological activity tests, the insecticidal activity of all synthesized derivatives **7a**–**7h** against common agricultural pests (*M. separata*, *M. persicae* and *R. padi*) were evaluated in the laboratory. The representative commercial insecticides chlorpyrifos, thiamethoxam and flunicotamid were used as positive controls, respectively. The target compounds **7a**–**7h** were also evaluated for acetylcholinesterase inhibitory activity in vitro.

#### 2.2.1. Acetylcholinesterase (AChE) Assay (in vivo)

Organophosphate compounds are inhibitors of AChE [24], and nereistoxin is also proven to be a weak AChE inhibitor [25]. Thus, the cholinesterase inhibitory activity of the synthesized derivatives **7a**–**7h** was evaluated spectrophotometrically against AChE (human) in vitro using the modified Ellman’s method [26,27]. The results are summarized in Table 1.

The inhibition rates of the compounds exceeded 90% within five minutes. The inhibition was maintained for a while and increased to more than 94% within 30 min, except for **7g**. The nereistoxin inhibition of AChE was only 66.02% at 5 min, while the inhibitory effect weakened as time elapsed. The inhibition rate was reduced to 41.27% after 30 min, confirming that nereistoxin is a weak inhibitor of AChE. Compounds **7a**–**7g** exhibited good inhibitory effects on AChE. Based on the average inhibition rates of the compounds listed in Table 1, **7a**–**7h** were chosen for further bioassays. The IC_50_ and K*_i_* values of the compounds for AChE were calculated (Table 2).

The AChE inhibition IC_50_ values ranged from 3.332 to 179.3 µM for the synthesized compounds, except for the unstable compounds **7f** and **7g** with poor solubility (Table 2). Compounds **7a** and **7b** produced high inhibition, with IC_50_ values of 3.3 μM and 3.4 μM, respectively. The IC_50_ values gradually increased from **7a** to **7e**, which suggests that the AChE inhibitory activity decreased as the substituted alkyl chain of phosphorothioate increased. The steric hindrance of various phosphate groups had a bigger influence on the inhibitory activity of AChE, and the lower the steric hindrance of the group, the stronger the inhibitory activity of AChE, according to a thorough analysis of the IC_50_ values for **7a**–**7h**.

#### 2.2.2. Insecticidal Activity against *M. separata*

The insecticidal activity of NTX and its derivatives **7a**–**7h** against the pre-third-instar larvae of *M. separata* was tested via the topical application method at a concentration of 2 mg/mL. Chlorpyrifos was used as the positive control at a concentration of 50 μg/mL. Most compounds showed excellent insecticidal bioactivity against *M. separata* at 24 h and 48 h (Table 3). The mortality values at 24 h and 48 h produced by **7a**, **7b**, **7c**, **7f** and **7g** at concentrations of 2 mg/mL were all 100%. It is notable that the insecticidal activity of nereistoxin against *M. separate* was also 100%. This indicates that *M. separate* can be effectively controlled by synthetic chemicals and nereistoxin in a short amount of time.

Based on these preliminary results (Table 3), the compounds with superior bioactivity were chosen for further bioassays against *M. separata* at different concentrations (The different concentrations of **7a**, **7b** and **7c** were 125, 250, 500, 1000 and 2000 μg/mL; those of 7**f** and 7**g** were 31.25, 62.5, 125, 250 and 500 μg/mL. Because of the high concentrations used, all the tested larvae died, making it impossible to calculate LC_50_ values.) The results of these bioassays are presented as half of the lethal concentration (LC_50_; μg/mL) in Table 4. The compounds used in the advanced bioassays were **7a**, **7b**, **7c**, **7f**, **7g** and nereistoxin. These compounds displayed substantial biological activity in the laboratory against *M. separata*, with LC_50_ values ranging from 136.86 to 836.34 μg/mL (Table 4). From the 95% confidence interval (CI), compound **7f** showed the highest biological activity, with an LC_50_ value of 136.86 μg/mL, followed by NTX, with an LC_50_ value of 150.71 μg/mL. NTX was slightly less active than **7f** but more active than other synthesized derivatives. The LC_50_ value of the positive control chlorpyrifos was only 16.31 μg/mL, indicating that the insecticidal activity of the synthesized derivative compounds against *M. separata* was much lower than that of chlorpyrifos. The LC_50_ values of **7f** and **7g**, however, were smaller than those of **7a**, **7b** and **7c**, demonstrating that compounds **7f** and **7g** have superior insecticidal activity against *M. separata* compared to the other compounds. Additionally, the steric hindrance of groups on **7f** and **7g** phosphate esters was relatively high, suggesting that enhancing the steric hindrance of groups on phosphate esters can enhance the compound’s insecticidal activity against *M. separata*.

#### 2.2.3. Insecticidal Activity against *M. persicae* Sulzer and *R. padi*

The insecticidal activity of NTX derivatives **7a**–**7h** against *M. persicae* and *R*. *padi* were tested using the slide-dip method and leaf-dipping method at a concentration of 0.2 mg/mL. Thiamethoxam and flunicotamid were used as positive controls at concentrations of 40 μg/mL. The corresponding mortality rates caused by these compounds at 48 h post-treatment were generally higher than those at 24 h (Table 5). For example, the corresponding mortality rates of **7b** against *M. persicae* and *R*. *padi* after 24 h were 20.65% and 30.20%, respectively. However, the corresponding mortality rates were 57.38% and 80.57% after 48 h, which were nearly 2–3 times greater than those after 24 h. Compound **7f** possessed the greatest bioactivity of the tested compounds. The corresponding mortality rates of **7f** against *M. persicae* and *R*. *padi* at 24 h were 13.03% and 19.92%, respectively. However, after 48 h, the corresponding mortality rates of **7f** sharply increased to 80.03% and 89.97%, which were almost 4.5–6 times higher than those after 24 h. These data suggest that the insecticidal effects of the compounds on both aphids can be effective in the short term. In contrast, the corresponding mortality rates against *M. persicae* and *R*. *padi* produced by NTX were at a moderate level among the tested compounds, with the corresponding mortality rates at 48 h being 34.19% and 40.22%, respectively.

Based on these preliminary results (Table 5), **7b** and **7f** were used, at different concentrations, for further bioassays against *M. persicae* and *R. padi*. The concentrations tested were 12.5, 25, 50, 100 and 200 μg/mL, and the results (LC_50_; μg/mL) are shown in Table 6 and Table 7.

The LC_50_ values of **7b** against *M. persicae and R. padi* were 42.93 μg/mL and 58.19 μg/mL, respectively, while the **7f** values were 138.37 μg/mL and 131.64 μg/mL (Table 6 and Table 7). These data indicate that **7b** was more toxic to the two aphids than **7f**, although Table 5 shows that the corresponding mortality rate of **7f** against *M. persicae* at 48 h was much higher than **7b**. The positive control LC_50_ value of thiamethoxam against *M. persicae* was 11.73 μg/mL, and the LC_50_ of flunicotamid against *R. padi* was 10.83 μg/mL. These values were both less than the LC_50_ values corresponding to **7f**. It is suggested that the insecticidal activity of these derivatives against *M. persicae* and *R. padi* is lower than that of thiamethoxam and flunicotamid. The LC_50_ values of **7b** against both aphids were lower than those of **7f**, indicating that **7b** has stronger insecticidal activity against both aphids than **7f**. The steric hindrance of the substituent group on phosphate ester of compound **7b** was smaller than that of **7f**, suggesting that the compound was more active with less steric hindrance in the substituent group, which is consistent with the compounds’ inhibition of AChE. In other words, the compound’s anti-aphid effect is directly tied to its inhibition of AChE.

### 2.3. Structural Analysis of Docking

Since thiophosphates exert insecticidal effects by inhibiting the activity of acetylcholinesterase, while nereistoxin insecticides bind mainly to acetylcholine receptors (AchR), docking simulations were performed to describe the selective behavior of the compounds at the binding site using acetylcholinesterase and Alpha7 nicotinic acetylcholine receptor (α7-nAChR) as target proteins. After optimizing the structures of **7a**–**7h**, they were used as small-molecule ligands, and two protein targets were used as acceptors. The interaction mode of the compounds and target proteins was analyzed to obtain the interactions between the compounds and the protein residues. The interactions could involve hydrogen bonding, π-π interaction and hydrophobic interactions. We referred to the docking score of the compounds with two acceptors to evaluate whether the synthesized compounds had biological activity. The molecular docking results are shown in Table 8.

The compounds were molecularly docked with two target proteins, respectively. Most compounds had a good binding effect with the two target proteins and had a high degree of matching (binding energy less than −6 kcal/mol). The binding energies of the compounds to the AChE site were lower than that of α7-nAchR, indicating that the compounds were more easily bound with acetylcholinesterase (Table 8). The complexes formed between the docking compounds and protein were visualized using Pymol 2.1 software (Schrödinger, New York, NY, USA)(the compound with the most negative binding energy was selected for each target), and the binding mode between the compound and protein was obtained. According to the binding mode, the binding amino acid residues of the compound and protein pockets were seen. Based on the results of compound substituents and acetylcholinesterase inhibitory activity, we selected **7b** (alkyl substituents) and **7g** (aryl substituents) for further analysis.

**Figure 2:**
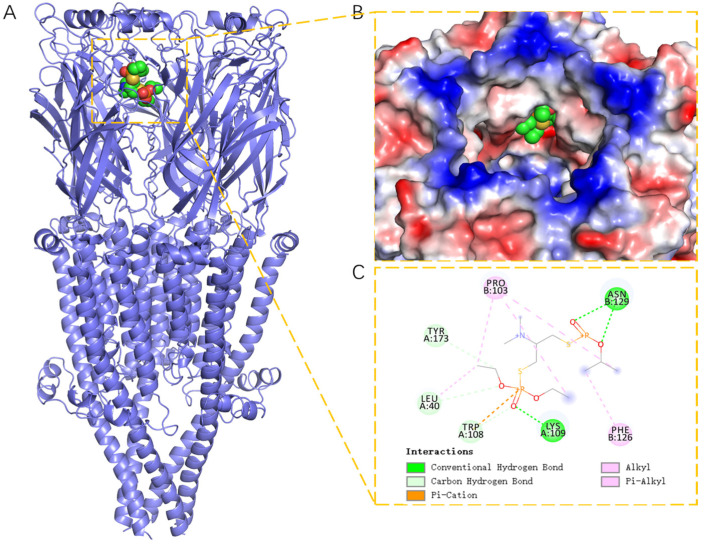
The binding mode of α7-nAchR with **7b**. (**A**) The 3D structure of the complex, (**B**) the hydrogen bond donor receptor network of the complex, (**C**) the 2D binding mode of the complex.

Regarding compound **7b**, a strong hydrophobic interaction was formed between the O atoms of **7b** and the amino acids (PRO-103, PHE-126) at the active site of α7-nAchR (Figure 2). This interaction plays an important role in stabilizing small molecules in the protein cavity. Also, strong hydrogen bond interactions were formed between the O atoms of **7b** and the amino acids (ASN-129, LYS-109) of α7-nAchR. Weak hydrogen bonds also existed between **7b** and amino acids (TYR-173, LEU-40, TRP-108). Furthermore, TRP-108 was also able to form a cation-π conjugation with **7b**. These interactions make an important contribution to anchoring small molecules in the protein cavity.

**Figure 3:**
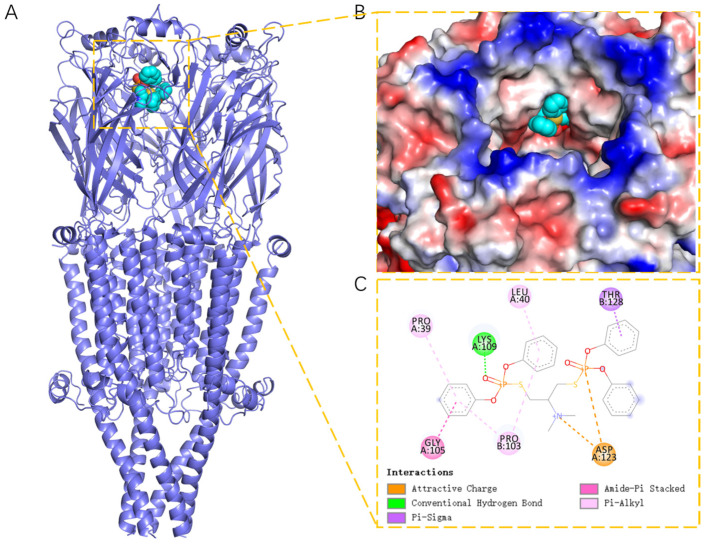
The binding mode of α7-nAchR with **7g**. (**A**) The 3D structure of the complex, (**B**) the hydrogen bond donor receptor network of the complex, (**C**) the 2D binding mode of the complex.

The amino acids of α7-nAchR that can interact with **7g** mainly include PRO-39, GLY-105, PRO-103, LEU-40, THR-128 and ASP-123 (Figure 3). Compound **7g** was well matched with the active site of α7-nAchR. It was able to form three types of interaction with the amino acids of α7-nAchR: a strong hydrogen bond between the O atoms of **7g** and LYS-109, hydrophobic interactions in PRO-39, PRO-103, LEU-40 and **7g**, and conjugate interactions of GLY-105, ASP-123 and THR-128 with **7g**. It is these interactions that effectively promote the formation of stable complexes between small molecules and α7-nAchR (Figure 3).

**Figure 4:**
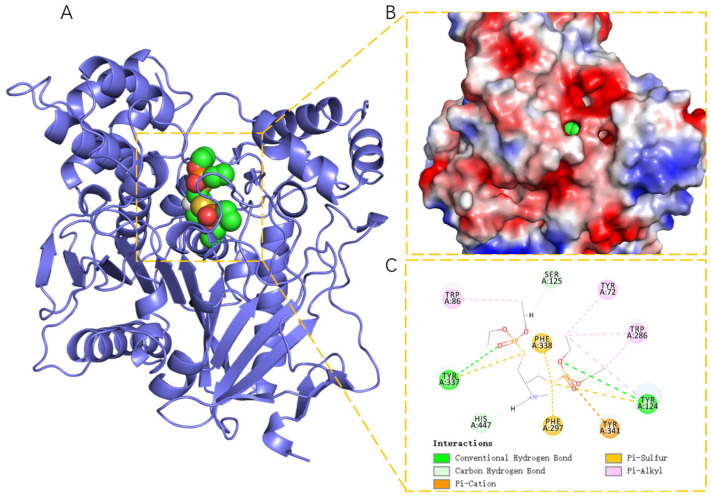
The binding mode of AChE with **7b**. (**A**) The 3D structure of the complex, (**B**) the hydrogen bond donor receptor network of the complex, (**C**) the 2D binding mode of the complex.

Compound **7b** was well matched with AChE. Strong hydrogen bonds were formed in the O atoms of **7b** and the amino acids (TYR-337, TYR-124) of AChE, which assisted in the stabilization of small molecules in protein pockets (Figure 4). Hydrophobic interactions also existed between **7b** and amino acids, such as TRP-86, TYR-72 and TRP-286. Furthermore, conjugated interactions of the amino acids PHE-338, PHE-297 and TYR-341 with **7b** were created, which are also helpful for stabilizing small molecules.

**Figure 5:**
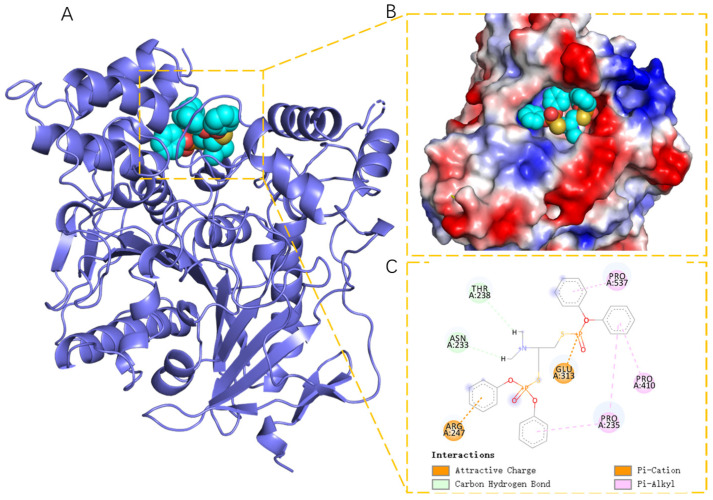
The binding mode of AChE with **7g**. (**A**) The 3D structure of the complex, (**B**) the hydrogen bond donor receptor network of the complex, (**C**) the 2D binding mode of the complex.

Concerning compound **7g**, it is well matched with AChE. There were hydrogen bonds formed between **7g** and the amino acids THR-238 and ASN-233. Hydrophobic interactions were also observed between the phenyl rings of compound **7g** and the amino acids PRO-537, PRO-410 and PRO-235. Conjugated interactions were created in **7g** with the amino acids ARG-247 and GLU-313 (Figure 5). All of these interactions can effectively promote the formation of a stable complex between **7g** and AChE.

Docking analysis revealed that the compounds were more likely to bind with AChE compared with AchR. Increasing hydrogen bonds and hydrophobic interactions with the active site of AChE could easily cause more potent inhibition in AChE. Hydrogen bonds were mainly formed between the O atom of the phosphate esters and the amino acid residues of AChE. Both alkyl and aryl groups can have hydrophobic interactions with the amino acid residues. Therefore, subsequent compounds can be designed to enrich the hydrogen binding sites, such as modifying phosphate esters with -COOH, -OH, -NH_2_.

## 3. Experimental Section

### 3.1. Chemistry

#### 3.1.1. General Procedures

All solvents and reagents were purchased from commercial sources and used without further purification. TLC analysis was performed on pre-coated, glass-backed silica gel plates and visualized using UV light and KMnO_4_. High-resolution mass spectra (HRMS) were obtained via Agilent 1290/6545 UHPLC-QTOF/MS. Tetramethylsilane was used as the internal standard. A Bruker 300M spectrometer was used to record ^1^H, ^13^C and ^31^P nuclear magnetic resonance (NMR) spectra in chloroform (CDCl_3_) and deuteroxide (D_2_O). Melting points were determined using METTLER TOLEDO MP90 Melting Point System.

#### 3.1.2. General Procedure for the Preparation of **2**

Compound **2** was synthesized via *N*-methylation of the Eschweiler–Clarke reaction. Formic acid (46.97 g, 98%, 1 mol) and 2-amino-1,3-propanediol (9.1 g, 0.1 mol) were added to a 250 mL round-bottom flask under ice bath conditions, and then, formaldehyde solution (aq., 37%, 18.4 g, 0.22 mol) was added dropwise to the above system. The reaction was stirred at 60 °C for 3 h until no additional bubbles were generated; then, the temperature was increased to 90 °C to continue the reaction for 6 h. After the reaction was completed, the excess formic acid and water were evaporated under reduced pressure to obtain crude 2-dimethylamino-1,3-propanediol **2** (10.35 g), which was a light-yellow oil.

#### 3.1.3. General Procedure for the Preparation of **3**

To a 25 mL round-bottom flask, crude compound **2** (2 g) was added, and SOCl_2_ (6.1 mL, 0.1 mol) was dropped slowly into an ice bath, and then, stirred under reflux for 0.5 h. Excess SOCl_2_ was removed and 1,3-dichloro-*N*,*N*-dimethyl-2-propanamine hydrochloride **3** (2.26 g) was obtained via recrystallization from chloroform.

*Data for* **3**. White solid, m.p.: 93.5 °C, yield 70%. ^1^H NMR (300 MHz, D_2_O) δ 4.03 (dt, *J* = 12.4, 5.4 Hz, 5H), 3.00 (s, 6H).^13^C NMR (75 MHz, CDCl_3_) δ 64.95 (s), 41.86 (s), 39.01 (s). MS (HRMS-ESI): Calcd for C_5_H_11_Cl_2_N, [M+H]^+^: 156.0341, found: 156.0327.

#### 3.1.4. General Procedure for the Preparation of **5**

Some phosphite diesters were obtained from commercial sources and others were synthesized according to previously reported procedures [28]. To a solution of the corresponding alcohol (0.3 mol) and pyridine (15.82 g, 0.2 mol) in Et_2_O (50 mL) at 0 °C was added PCl_3_ (13.74 g, 0.1 mol) over the course of 1 h. After complete addition, the reaction mixture was allowed to slowly warm to ambient temperature, and it was stirred for 16 h. The white suspension was then filtered under suction, and the residual pyridinium chloride was washed twice with Et_2_O (50 mL). The filtrates were concentrated under reduced pressure and dried under reduced pressure to yield the desired phosphonates, which were colorless liquids.

#### 3.1.5. General Procedure for the Preparation of **6**

Compounds **6a**–**6h** were synthesized based on information from the literature [28]. To a suspension of the appropriate phosphonate (20 mmol) and S_8_ (0.704 g, 22 mmol) in Et_2_O in a round-bottom flask was slowly added NEt_3_ (2.23 g, 22 mmol) in an ice bath. After the full conversion of the phosphonate, as monitored via ^31^P NMR spectroscopy, the suspension was diluted with Et_2_O to 100 mL, and then, washed with aqueous HCl (100 mL, 1 M), dried over MgSO_4_ and concentrated under reduced pressure. The resulting suspension was filtered to yield *S*-hydrogen phosphorothioates.

#### 3.1.6. General Procedure for the Preparation of **7a**–**7h**

A mixture of *S*-hydrogen phosphorothioates **6a**–**6h** (3 mmol) and NaH (60% in mineral oil, 0.12 g, 3 mmol) in dry acetonitrile (10 mL) was stirred at room temperature. A quantity of 1,3-dichloro-*N*,*N*-dimethyl-2-propanamine hydrochloride **3** (0.193 g, 1 mmol) was added after 10 min, and the reaction was stirred at 50 °C, as monitored via TLC. After completion, the reaction mixture was filtered and the organic phase concentrated in vacuo. The crude product was purified via silica gel column chromatography to give compounds **7a**–7**h** in 20–78% yields (see Supplementary Materials).

*Data for* **7a**. Yellow oil, yield 26% (silica gel chromatography: ethyl acetate/methanol = 20:1, R*_f_* = 0.16). ^31^P NMR (121 MHz, CDCl_3_) δ 31.10 (s). ^1^H NMR (300 MHz, CDCl_3_) δ 3.79 (d, *J* = 12.6 Hz, 12H), 3.12–2.83 (m, 5H), 2.31 (s, 6H). ^13^C NMR (75 MHz, CDCl_3_) δ 64.68 (t, *J* = 5.3 Hz), 54.01 (d, *J* = 6.1 Hz), 40.20 (s), 30.17 (d, *J* = 3.4 Hz). MS (HRMS-ESI): Calcd for C_9_H_23_NO_6_P_2_S_2_, [M+H]^+^: 368.0515, found: 368.0536.

*Data for* **7b**. Yellow oil, yield 54% (silica gel chromatography: petroleum ether/ethyl acetate = 1:2, R*_f_* = 0.20). ^31^P NMR (121 MHz, CDCl_3_) δ 27.66 (s). ^1^H NMR (300 MHz, CDCl_3_) δ = 4.34–4.04 (m, 8H), 3.27–2.71 (m, 5H), 2.34 (s, 6H), 1.52–1.28 (m, 12H). ^13^C NMR (75 MHz, CDCl_3_) δ 64.61 (t, *J* = 5.7 Hz), 63.72 (d, *J* = 6.2 Hz), 40.25 (s), 30.24 (d, *J* = 3.4 Hz), 16.13 (d, *J* = 7.2 Hz). MS (HRMS-ESI): Calcd for C_13_H_31_NO_6_P_2_S_2_, [M+H]^+^: 424.1141, found: 424.1177.

*Data for* **7c**. Yellow oil, yield 74% (silica gel chromatography: petroleum ether/ethyl acetate = 1:6, R*_f_* = 0.18). ^1^P NMR (121 MHz, CDCl_3_) δ 27.82 (s). ^1^H NMR (300 MHz, CDCl_3_) δ 4.20–3.94 (m, 8H), 3.18–2.86 (m, 5H), 2.34 (s, 6H), 1.88–1.62 (m, 8H), 0.98 (t, *J* = 7.4 Hz, 12H). ^13^C NMR (75 MHz, CDCl_3_) δ 69.16 (d, *J* = 5.9 Hz), 64.54 (t, *J* = 5.9 Hz), 40.27 (s), 30.19 (d, *J* = 3.3 Hz), 23.58 (d, *J* = 7.3 Hz), 10.12 (s). MS (HRMS-ESI): Calcd for C_17_H_39_NO_6_P_2_S_2_, [M+H]^+^: 480.1767, found: 480.1791.

*Data for* **7d**. Yellow oil, yield 61% (silica gel chromatography: petroleum ether/ethyl acetate = 3:2, R*_f_* = 0.22). ^31^P NMR (121 MHz, CDCl_3_) δ 25.23 (s). ^1^H NMR (300 MHz, CDCl_3_) δ 4.85–4.63 (m, 4H), 3.16–2.85 (m, 5H), 2.34 (s, 6H), 1.37 (t, *J* = 5.8 Hz, 24H). ^13^C NMR (75 MHz, CDCl_3_) δ 72.71 (d, *J* = 6.5 Hz), 64.37 (t, *J* = 6.4 Hz), 40.22 (s), 30.41 (d, *J* = 3.3 Hz), 24.08–23.55 (m). MS (HRMS-ESI): Calcd for C_17_H_39_NO_6_P_2_S_2_, [M+H]^+^: 480.1767, found: 480.1796.

*Data for* **7e**. Yellow oil, yield 78% (silica gel chromatography: petroleum ether/ethyl acetate = 1:3, R*_f_* = 0.25). ^31^P NMR (121 MHz, CDCl_3_) δ 27.80 (s). ^1^H NMR (300 MHz, CDCl_3_) δ 4.23–3.95 (m, 8H), 3.21–2.79 (m, 5H), 2.31 (s, 6H), 1.78–1.57 (m, 8H), 1.50–1.31 (m, 8H), 0.92 (t, *J* = 7.4 Hz, 12H). ^13^C NMR (75 MHz, CDCl_3_) δ 67.42 (dd, *J* = 6.5, 0.9 Hz), 64.54 (t, *J* = 5.9 Hz), 40.28 (s), 32.19 (d, *J* = 7.2 Hz), 30.21 (d, *J* = 3.3 Hz), 18.77 (s), 13.63 (s). MS (HRMS-ESI): Calcd for C_21_H_47_NO_6_P_2_S_2_, [M+H]^+^: 536.2393, found: 536.2429.

*Data for* **7f**. Yellow oil, yield 30% (silica gel chromatography: petroleum ether/ethyl acetate = 1:6, R*_f_* = 0.22). ^31^P NMR (121 MHz, CDCl_3_) δ 17.31 (s). ^1^H NMR (300 MHz, CDCl_3_) δ 3.15–2.81 (m, 5H), 2.34 (s, 6H), 1.55 (s, 36H). ^13^C NMR (75 MHz, CDCl_3_) δ 84.60 (dd, *J* = 9.5, 1.5 Hz), 63.90 (t, *J* = 6.5 Hz), 40.43 (s), 30.85 (d, *J* = 3.7 Hz), 30.30 (d, *J* = 4.3 Hz). MS (HRMS-ESI): Calcd for C_21_H_47_NO_6_P_2_S_2_, [M+H]^+^: 536.2393, found: 536.2424.

*Data for* **7g**. White oil, yield 26% (silica gel chromatography: petroleum ether/ethyl acetate = 3:1, R*_f_* = 0.32). ^31^P NMR (121 MHz, CDCl_3_) δ 21.40 (s). ^1^H NMR (300 MHz, CDCl_3_) δ 7.43–7.10 (m, 20H), 3.15–2.74 (m, 4H), 2.68–2.44 (m, 1H), 2.13 (s, 6H). ^13^C NMR (75 MHz, CDCl_3_) δ 150.25–149.92 (m), 129.92 (d, *J* = 1.0 Hz), 125.77 (s), 120.76 (dd, *J* = 7.9, 4.9 Hz), 64.19 (t, *J* = 5.8 Hz), 40.01 (s), 31.10 (d, *J* = 3.6 Hz). MS (HRMS-ESI): Calcd for C_29_H_31_NO_6_P_2_S_2_, [M+H]^+^: 616.1141, found: 616.1153.

*Data for* **7h**. Yellow oil, yield 20% (silica gel chromatography: petroleum ether/ethyl acetate = 1:2, R*_f_* = 0.15). ^31^P NMR (121 MHz, CDCl_3_) δ 28.76 (s). ^1^H NMR (300 MHz, CDCl_3_) δ 7.35 (d, *J* = 5.9 Hz, 20H), 5.24–4.99 (m, 8H), 3.05–2.63 (m, 5H), 2.14 (s, 6H). ^13^C NMR (75 MHz, CDCl_3_) δ 135.46 (d, *J* = 7.4 Hz), 128.67 (s), 128.18 (s), 69.08 (d, *J* = 6.0 Hz), 64.07 (t, *J* = 6.0 Hz), 40.09 (s), 30.25 (d, *J* = 3.4 Hz). MS (HRMS-ESI): Calcd for C_33_H_39_NO_6_P_2_S_2_, [M+H]^+^: 672.1767, found: 672.1733.

### 3.2. Biological Tests

#### 3.2.1. Bioassay Methods

All of the bioassays were performed on representative test insects reared in the laboratory. Bioassays were repeated in triplicate. Insecticidal assessments were made on a dead/alive basis, and mortality rates were corrected using Abbott’s formula.

#### 3.2.2. Inhibitory Activity of Acetylcholinesterase (AChE)

In vitro AChE inhibitory activity of the synthesized derivatives was determined spectrophotometrically against AChE using the modified Ellman’s method. The solutions and concentrations involved in the assay were as follows: phosphate buffer (0.1 M, Na_2_HPO_4_/NaH_2_PO_4_, pH = 7.4), the enzyme (0.4 μg/mL in phosphate buffer, pH = 7.4), DTNB (5,5-dithiobis (2-nitrobenzoic acid)) (1.5 mmol/L concentration) and ATCh (acetylthiocholine iodide) (2.25 mmol/L concentration). The compounds were dissolved in dimethyl sulfoxide (DMSO, 99%, Macklin, Shanghai, China) and configured as a stock solution at a concentration of 200 mmol/L. The solutions were then added to the buffer. The maximum concentration of DMSO in the assays was 2%, a concentration that had no effect on inhibitory activity in independent experiments without the inhibitor. The absorbance at 412 nm was determined at 37 °C. It was continuously measured for 30 min, and each measurement interval was 1 min. The linear reaction part was taken for the kinetic calculation. The same amount of PB buffer solution replaced the test sample as the blank control. It was considered that the absorbance change was proportional to the enzyme concentrations in principle. The IC_50_ values were determined via probit analysis using GraphPad Prism 8.3 software (GraphPad Software, San Diego, CA, USA).

The formulas involved in the experiment were as follows:

Inhibitory mortality rate (%) = (B_412_ − T_412_) × 100/B_412_

where B_412_ is the absorbance at 412 nm of the blank control, and T_412_ is the absorbance at 412 nm of the treated group.

*K_i_* = ln[*v*_0_/*v_t_*]/[IN]*t*

where [IN] is the initial concentration of the tested compound, and *v*_0_ and *v_t_* are the reaction rates at time zero and time *t*, respectively [29].

#### 3.2.3. Insecticidal Activity against *M. separata*

The insecticidal activity of the synthesized compounds was evaluated in the laboratory using a topical application method [30]. Fresh, tender corn leaves were cut into squares with a side length of about 0.5 cm. Two drops of the sample solution were added to the leaves with a 1 μL micro dropper, and the operation was repeated for different concentrations of the sample solution. The treated leaves were air dried and then placed into the insect-rearing box. Ten pre-third-instar larvae of *M. separata* were raised in the insect box, and ten leaves were placed in each box. The test was repeated 4 times, with acetone as the blank control and 97% chlorpyrifos as the positive control. The experiment was carried out at 25 ± 2 °C and a relative humidity (RH) of 65−80%, and over a 12 h/12 h (light/dark) photoperiod. The number of survivors and deaths were recorded after 24 h and 48 h. The insecticidal activity of the tested compounds against the pre-third-instar larvae of *M. separata* was calculated using the formula:

Corrected mortality rate (%) = (T − C) × 100/(1 − C)

where T is the mortality rate in the treated group expressed as a percentage, and C is the mortality rate in the untreated group expressed as a percentage.

The LC_50_ values were determined via probit analysis using IBM SPSS Statistics software (IBM, New York, NY, USA).

#### 3.2.4. Insecticidal Activity against *M. persicae*

The insecticidal activity of the synthesized compounds against *M. persicae* was evaluated in the laboratory using the slide-dip method [31]. The compounds were dissolved in acetone, and then, diluted in 0.1% Tween-80 aqueous solution to final concentrations ranging from 0.125 to 2 mg/mL; the diluent (0.1% Tween-80) alone served as a blank control, and 98% thiamethoxam was the positive control. Adult aphids were fixed on double-sided tape on slides using a small brush. One end of the slide with the aphids was immersed in the test solutions for 5 s. The excess liquid was removed using absorbent paper, and the mortality rates were calculated at 24 h and 48 h after treatment. Each test was repeated three times with 30 adult aphids each time.

#### 3.2.5. Insecticidal Activity against *R. padi*

The insecticidal activity of the synthesized compounds against *R. padi* was evaluated in the laboratory using the leaf-dipping method [32]. The compounds were dissolved in acetone, and then, diluted in 0.1% Tween-80 aqueous solution to final concentrations ranging from 12.5 to 200 μg/mL; the diluent (0.1% Tween-80) alone served as a blank control, and 96% flunicotamid was the positive control. Wheat leaves were cut into 2–5 cm sections (3–5 leaves for each concentration), immersed into different concentrations of sample solution for 3–5 s, and then, taken out. The excess liquid on the leaves was removed using filter paper, and the leaves were air dried. A total of 20–50 aphids were raised on each leaf at room temperature. The test was repeated 4 times for each concentration. The mortality and survival rates at 24 h and 48 h were recorded.

### 3.3. Molecular Docking

#### 3.3.1. Preparation of Target Proteins and Small-Molecular Structures

The crystal structures of AChE (PDB ID: 6WVO) and a7-nAchR (PDB ID: 7EKP) target proteins came from the protein database (https://www.rcsb.org/ (accessed on 31 May 2023)). After the irrelevant small molecules in the protein molecule were deleted by Pymol 2.1 software (Schrödinger, New York, NY, USA), the protein molecule was guided to AutoDock Tools 1.5.6 software (Scripps Institute, San Diego, CA, USA) to delete water molecules, add hydrogen atoms and set the atomic type. It was saved as a pdbqt file.

The structures of **7a**–**7h** were constructed using ChemDraw (CambridgeSoft, Cambridge, MA, USA). The energies of the downloaded compounds were minimized using Chem3D and converted into mol^2^ format. The small-molecule compounds were imported into AutoDock Tools 1.5.6 software (Scripps Institute, San Diego, CA, USA), their atomic charges were added and their atomic types were assigned. All the flexible keys were rotated by default, and it was finally saved as a pdbqt file.

#### 3.3.2. Analysis of Molecular Docking

The treated compounds were used as small-molecule ligands and two protein targets were used as acceptors. The center position and length, width and height of the Grid Box were determined based on the interaction between small molecules, and the targets were set to 40 Å × 40 Å × 40 Å. AutoDock Vina was used to conduct batch molecular docking, and the results of molecular docking were analyzed. Pymol 2.1 software (Schrödinger, New York, NY, USA) was used to visualize interactions between the compounds and proteins. The final docking structure was evaluated according to the binding free energy.

## 4. Conclusions

In this study, a class of ring-opened nereistoxin derivatives modified with phosphate esters was synthesized based on *S*-hydrogen phosphorothioates and 2-dimethylamino-1,3-dichloropropane, and characterized via ^1^H, ^13^C and ^31^P NMR and HRMS. The biological activity of the compounds was assessed based on the inhibition of AChE and insecticidal activity of armyworms and aphids. The majority of substances demonstrated potent AChE inhibition in a short time (within 5−30 min). The enzyme inhibitory effect of compounds was closely related to the steric hindrance of the substituted groups on phosphate. The smaller the steric hindrance, the stronger the inhibitory activity of AChE. That is to say, **7a** had the strongest enzyme inhibitory effect, with a minimum IC_50_ value of 3.332 μM. Compounds **7a**, **7b**, **7c**, **7f** and **7g** showed the most effective insecticidal activity against armyworm among these derivatives, with a 24 h final mortality rate of 100%. Compounds **7b** and **7f** showed greater insecticidal efficacy against *M. persicae* and *R. padi*, with a maximum mortality of 90% after 48 h. These findings imply that pests of the hemiptera and lepidopteran families are both susceptible to the insecticidal effects of phosphonate-modified nereistoxin derivatives. The results of this study may help to expand the use of nereistoxin insecticides and contribute to the development of new insecticide formulations. Additionally, molecular docking was performed to analyze the selectivity of compounds for binding to AchR or AChE. The results demonstrated that the compounds had a higher propensity to bind with AChE. The interactions between the chemicals and AChE were made more effective by hydrogen bonds and hydrophobic interactions.

## Figures and Tables

**Table 1:** Average inhibition rates of NTX and compounds **7a**–**7h** on AChE at 2 mmol/L (*n* = 3).

Compound	R	AChE Inhibition Rate (%)		
		5 min	20 min	30 min
**7a**	Me	90.78	96.10	96.26
**7b**	Et	91.90	96.82	97.11
**7c**	*^n^*Pr	92.71	97.13	97.39
**7d**	*^i^*Pr	92.73	97.10	97.43
**7e**	*^n^*Bu	92.87	97.21	97.48
**7f**	*^t^*Bu	89.56	94.58	94.51
**7g**	Ph	72.84	66.73	60.64
**7h**	Bn	92.59	97.08	97.35
Nereistoxin	/	66.02	53.98	41.27

**Table 2:** Experimental IC_50_ and K*_i_* values of all synthesized compounds pertinent to AChE.

Compound	R	IC_50_ (μM)	Final Concentration (μM) *^a^*	K*_i_* (mM^−1^⋅min^−1^)
**7a**	Me	3.332	3.125	115.86 ± 64.99
**7b**	Et	3.422	3.125	83.83 ± 3.12
**7c**	*^n^*Pr	10.28	12.5	33.95 ± 5.62
**7d**	*^i^*Pr	37.83	50	7.92 ± 3.73
**7e**	*^n^*Bu	179.3	200	0.94 ± 0.21
**7f**	*^t^*Bu	— *^b^*	—	—
**7g**	Ph	— *^c^*	—	—
**7h**	Bn	61.4	50	3.67 ± 0.14
Nereistoxin	/	— *^d^*	—	—

*^a^* The concentration of the compounds after adding them to the PBS buffer solution containing enzyme, substrate and chromogenic agent DTNB; *^b^* hydrolyzed easily; *^c^* poor solubility; *^d^* the inhibition rate of 2 mM NTX on AChE was 41.27% (30 min), and the IC_50_ value could not be calculated.

**Table 3:** Corrected mortality rate of compounds **7a**–**7h**, nereistoxin and positive controls (chlorpyrifos) against *M. separata* at 2 mg/mL.

Compound	R	Corrected Mortality Rate (%)	
		24 h	48 h
**7a**	Me	100	100
**7b**	Et	100	100
**7c**	*^n^*Pr	100	100
**7d**	*^i^*Pr	70	62.5
**7e**	*^n^*Bu	70	62.5
**7f**	*^t^*Bu	100	100
**7g**	Ph	100	100
**7h**	Bn	40	25
Nereistoxin	/	100	100
Chlorpyrifos	/	100	100

**Table 4:** LC_50_ values of potent compounds, nereistoxin and chlorpyrifos against *M. separata* in the laboratory.

Compound	y = ax + b	LC_50_ (μg/mL)	R^2^	CI (95%)
**7a**	y = 1.0818x + 1.8613	796.91	0.9796	336.36–1888.06
**7b**	y = 1.3741x + 0.9845	836.34	0.9583	408.44–1712.54
**7c**	y = 1.6902x + 0.0770	817.84	0.9653	448.10–1492.67
**7f**	y = 1.3767x + 2.0590	136.86	0.9864	73.56–254.45
**7g**	y = 1.7743x + 1.0230	174.33	0.9979	102.15–297.51
Nereistoxin	y = 1.8644x + 0.9391	150.71	0.9784	92.25–246.21
Chlorpyrifos	y = 1.6690x + 2.9764	16.31	0.9829	9.44–28.20

**Table 5:** Corrected mortality rates of compounds **7a**–**7h**, nereistoxin and positive controls (thiamethoxam and flunicotamid) against *M. persicae* and *R. padi* at 0.2 mg/mL.

Compound	R	Corrected Mortality Rate (%)			
		*M. persicae*		*R. padi*	
		24 h	48 h	24 h	48 h
**7a**	Me	21.71	32.71	20.86	44.56
**7b**	Et	20.65	57.38	30.20	83.57
**7c**	*^n^*Pr	23.16	33.45	21.32	42.43
**7d**	*^i^*Pr	15.51	17.68	24.63	27.50
**7e**	*^n^*Bu	42.93	40.25	43.01	43.12
**7f**	*^t^*Bu	13.03	80.03	19.92	89.97
**7g**	Ph	3.96	3.61	7.15	25.45
**7h**	Bn	−0.63	23.27	0.48	32.23
Nereistoxin	\	14.25	34.19	12.71	40.22
Thiamethoxam	\	70.01	94.12	—	—
Flunicotamid	\	—	—		93.23

**Table 6:** LC_50_ values of potent compounds and thiamethoxam against *M.persicae* in the laboratory.

Compound	*M. persicae*			
	y = ax + b	LC_50_ (μg/mL)	R^2^	CI (95%)
**7b**	y = 0.0014x + 0.4399	42.93	0.9540	41.37–67.58
**7f**	y = 0.0019x + 0.2371	138.37	0.9662	20.08–57.26
Thiamethoxam	y = 0.0194x + 0.2725	11.73	0.9684	21.80–77.11

**Table 7:** LC_50_ values of potent compounds and flunicotamid against *R. padi* in the laboratory.

Compound	*R. padi*			
	y = ax + b	LC_50_ (μg/mL)	R^2^	CI (95%)
**7b**	y = 1.6584x + 2.0732	58.19	0.9839	50.62–66.88
**7f**	y = 1.245x + 2.3602	131.64	0.9858	101.15–171.32
Flunicotamid	y = 2.2140x + 2.7094	10.83	0.9726	6.67–12.12

**Table 8:** Binding energies of compounds **7a**–**7h** to active sites.

Compound	R	AChE (kcal/mol)		α7-nAchR (kcal/mol)	
		AChE-1	AChE-2	α7-nAchR-1	α7-nAchR-2
**7a**	Me	−6.2	−6.1	−5.4	−4.8
**7b**	Et	−6.6	−6.4	−5.7	−4.9
**7c**	*^n^*Pr	−7.0	−7.0	−5.9	−5.0
**7d**	*^i^*Pr	−7.4	−6.9	−6.0	−5.5
**7e**	*^n^*Bu	−7.2	−5.0	−5.9	−5.0
**7f**	*^t^*Bu	−6.7	−5.4	−6.2	−5.6
**7g**	Ph	−9.2	−9.1	−8.1	−7.4
**7h**	Bn	−9.0	−6.2	−8.5	−6.5

## Supplementary Materials

The following supporting information can be downloaded at: https://www.mdpi.com/article/10.3390/molecules28124846/s1, Figure S1: ^31^P NMR of compound **7a**.; Figure S2: ^1^H NMR of compound 7**a**; Figure S3: ^13^C NMR of compound **7a**; Figure S4: HRMS of compound **7a**; Figure S5: ^31^P NMR of compound **7b**; Figure S6: ^1^H NMR of compound **7b**; Figure S7: ^13^C NMR of compound **7b**; Figure S8: HRMS of compound **7b**; Figure S9: ^31^P NMR of compound **7c**; Figure S10: ^1^H NMR of compound **7c**; Figure S11: ^13^C NMR of compound **7c**; Figure S12: HRMS of compound **7c**; Figure S13: ^31^P NMR of compound **7d**; Figure S14: ^1^H NMR of compound **7d**; Figure S15: ^13^C NMR of compound **7d**; Figure S16: HRMS of compound **7d**; Figure S17: ^31^P NMR of compound **7e**; Figure S18: 1H NMR of compound **7e**; Figure S19: ^13^C NMR of compound **7e**; Figure S20: HRMS of compound **7e**; Figure S21: ^31^P NMR of compound **7f**; Figure S22: ^1^H NMR of compound **7f**; Figure S23: ^13^C NMR of compound **7f**; Figure S24: HRMS of compound **7f**; Figure S25: ^31^P NMR of compound **7g**; Figure S26: ^1^H NMR of compound **7g**; Figure S27: ^13^C NMR of compound **7g**; Figure S28: HRMS of compound **7g**; Figure S29: ^31^P NMR of compound **7h**; Figure S30: ^1^H NMR of compound **7h**; Figure S31: ^13^C NMR of compound **7h**; Figure S32: HRMS of compound **7h**; Figure S33: The concentration-inhibition rate relationship of **7a**, **7b**, **7c**, **7d**, **7e** and **7h**; Figure S34: The concentration-corrected mortality rate (against *M. separata*) relationship of **7a**, **7b**, **7c**, **7f**, **7g**, nereistoxin and chlorpyrifos; Figure S35: The concentration-corrected mortality rate (against *M. persicae*) relationship of **7b**, **7f** and thiamethoxam; Figure S36: The concentration-corrected mortality rate (against *R. padi*) relationship of **7b**, **7f** and flunicotamid.

## References

[1] Emmel T.C., Scoble N.J. (1995). The Lepidoptera: Form, Function and Diversity.

[2] Sharma S., Kooner R., Arora R. (2017). Insect pests and crop losses. Breeding Insect Resistant Crops for Sustainable Agriculture.

[3] Chabert A., Sarthou J.P. (2017). Practices of conservation agriculture prevail over cropping systems and landscape heterogeneity in understanding the ecosystem service of aphid biocontrol. Agric. Ecosyst. Environ..

[4] Yi X.U., Gray S.M. (2020). Aphids and their transmitted potato viruses: A continuous challenges in potato crops. J. Integr. Agric..

[5] Naveen N.C., Chaubey R., Kumar D., Rebijith K.B., Rajagopal R., Subrahmanyam B., Subramanian S. (2017). Insecticide resistance status in the whitefly, Bemisia tabaci genetic groups Asia-I, Asia-II-1 and Asia-II-7 on the Indian subcontinent. Sci. Rep..

[6] Ribeiro L.M.S., Wanderley-Teixeira V., Ferreira H.N., Teixeira Á.A.C., Siqueira H.A.A. (2014). Fitness costs associated with field–evolved resistance to chlorantraniliprole in *Plutella xylostella* (Lepidoptera: Plutellidae). Entomol. Res..

[7] Lorsbach B.A., Sparks T.C., Cicchillo R.M., Garizi N.V., Hahn D.R., Meyer K.G. (2019). Natural products: A strategic lead generation approach in crop protection discovery. Pest Manage. Sci..

[8] Nitta S. (1934). Nereistoxin, a poisonous constituent of *Lumbriconereis heteropoda* Marenz (Eunicidae). Yakugaku Zasshi.

[9] Konishi K. (1968). Organic insecticides. Part X. Synthesis of nereistoxin and related compounds III. Agric. Biol. Chem..

[10] Konishi K. (1970). Organic insecticides. Part XI. Synthesis of nereistoxin and related compounds IV. Agric. Biol. Chem..

[11] Konishi K. (1970). Organic insecticides. Part XIII. Synthesis of nereistoxin andrelated compounds VI. Agric. Biol. Chem..

[12] Konishi K., Tahori A.S. (1972). Pesticide Chemistry: Proceedings of the Second International IUPAC Congress of Pesticide Chemistry.

[13] Gholivand K., Mohammadpanah F., Pooyan M., Valmoozi A.A.E., Sharifi M., Mani-Varnosfaderani A., Hosseini Z. (2019). Synthesis, crystal structure, insecticidal activities, molecular docking and QSAR studies of some new phospho guanidines and phospho pyrazines as cholinesterase inhibitors. Pestic. Biochem. Physiol..

[14] Wang X., Zhu Q., Yan X., Wang Y., Liao C., Jiang G. (2020). A review of organophosphate flame retardants and plasticizers in the environment: Analysis, occurrence and risk assessment. Sci. Total Environ..

[15] Li A., Fan Y., Cao X., Chen L., Wang L., Alves C.S., Mignani S., Majoral J.P., Tomas H., Shi X. (2019). Morpholino-functionalized phosphorus dendrimers for precision regenerative medicine: Osteogenic differentiation of mesenchymal stem cells. Nanoscale.

[16] Mchardy S.F., Wang H.Y.L., Mccowen S.V., Valdez M.C. (2017). Recent advances in acetylcholinesterase Inhibitors and Reactivators: An update on the patent literature (2012–2015). Expert Opin. Ther. Pat..

[17] Baran P., Knouse K.W., Huang Y., Qiu S., Mcdonald I. (2021). A P(V)-platform for oligonucleotide synthesis. Science.

[18] Oswaldo P., Nicolaas S., Martin B. (2021). Preparative Synthesis of an RP-Guanosine-3′,5′-Cyclic Phosphorothioate Analogue, a Drug Candidate for the Treatment of Retinal Degenerations. Org. Process Res. Dev..

[19] Crooke S.T., Bennett C.F. (1996). Progress in antisense oligonucleotide therapeutics. Annu. Rev. Pharmacol. Toxicol..

[20] Chang Z., Yu L., Xi Z., Lan M. (1998). Synthesis of Shacanlin Compounds.

[21] Yu L., Xi Z., Lan M., Chang Z. (1998). Synthesis and biological activity tests of Shacanlin. Pesticides.

[22] Zhao H., Yu L. (1998). Toxicity and control efficacy of O, O, O′, O′-Tetramethyl-S, S′-(2-*N*,*N*-dimethylamino-trimethylene)-bis-dithiophosphate against Wheat Aphid. Pesticides.

[23] Yurugi S., Sakai M., Kato M., Hagiwara H., Koniski K., Harukawa T. (1964). Dithio Amino Alkanes.

[24] Milan J. (2018). Neurotoxic effects of organophosphorus pesticides and possible association with neurodegenerative diseases in man: A review. Toxicology.

[25] Sakai M. (1966). Studies on the insecticidal action of nereistoxin.IV. Role of the anticholinesterase activity in the insecticideal action to housefly *Musca domestica* L. (Diptera: Musicidae). Appl. Ent. Zool..

[26] Ellman G.L., Courtney K.D., Andres V., Featherstone R.M. (1961). A new and rapid colorimetric determination of acetylcholinesterase activity. Biochem. Pharmacol..

[27] Olivera P., Ivana C., Anita V. (2019). Phytochemical composition, antiradical and anticholinesterase potentials of *Centaurea alba* and *Centaurea jacea* volatile oils. Croat. Chem. Acta..

[28] Santschi N., Togni A. (2011). Electrophilic trifluoromethylation of *S*-hydrogen phosphorothioates. J. Org. Chem..

[29] Franz W., Horst T., Ladislaus S., Eyer P. (2004). Kinetic analysis of interactions between human acetylcholinesterase, structurally different organophosphorus compounds and oximes. Biochem. Pharmacol..

[30] Sachiyo S., Masaya M. (2011). Effect of acetone solution in a topical application method on mortality of rice planthoppers, *Nilaparvata lugens*, *Sogatella furcifera*, and *Laodelphax striatellus* (Homoptera: Delphacidae). Appl. Entomol. Zool..

[31] Shi M., Wei J., Qi L., Tian L., Wen W., Hang B., Bao S. (2021). Design, Synthesis, and Study of the Insecticidal Activity of Novel Steroidal 1,3,4-Oxadiazoles. J. Agric. Food Chem..

[32] Che Z., Yu X., Zhi X., Fan L., Yao X., Xu H. (2013). Synthesis of novel 4α-(acyloxy)-2′(2′,6′)-(di)halogenopodophyllotoxin derivatives as insecticidal agents. J. Agric. Food Chem..

