# Supplementary material for: Synthesis, Bioactivity and Molecular Docking of Nereistoxin Derivatives Containing Phosphonate
Source: Molecules. 2023 Jun 19;28(12):4846. doi: 10.3390/molecules28124846 (PMC10305189; doi:10.3390/molecules28124846)
Supplement: Supplementary file 1 [file molecules-28-04846-s001.zip › molecules-2456597-supplementary.pdf]

molecules

Supporting Information

Synthesis, Bioactivities and Molecular Docking of Nereistoxin Derivatives Containing Phosphonate

Qiaoli Yan, Xiaogang Lu, Zixuan Zhang, Qian Jin, Runli Gao, Liqin Li * and Hongmei Wang *

State Key Laboratory of NBC Protection for Civilian, Beijing 102205, China; 17801202159@163.com (Q.Y.); luxg2018@sina.com (X.L.); zzxuan99599@163.com (Z.Z.); jinqian160@163.com (Q.J.); gaorunli@163.com (R.G.)

* Correspondence: llq969696@126.com (L.L.); hongmei_ricd@yeah.net (H.W.)

The ^{1}H-NMR, ^{13}C-NMR, ^{31}P-NMR spectra and HRMS of nereistoxin derivatives **7a-7h** were listed as below:

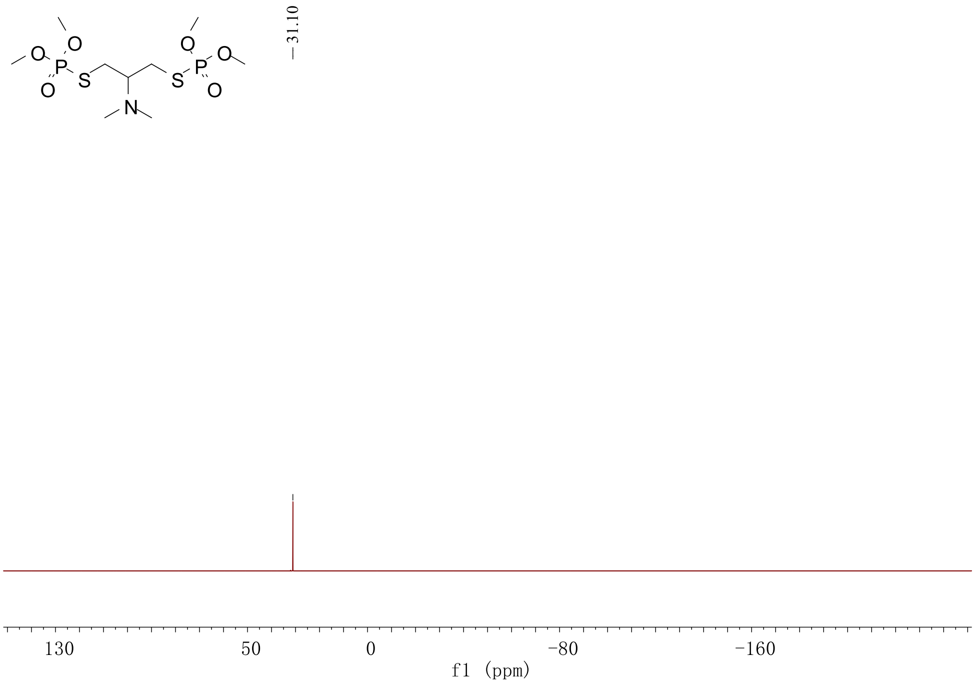

Figure S1. ^{31}P-NMR of compound **7a**.

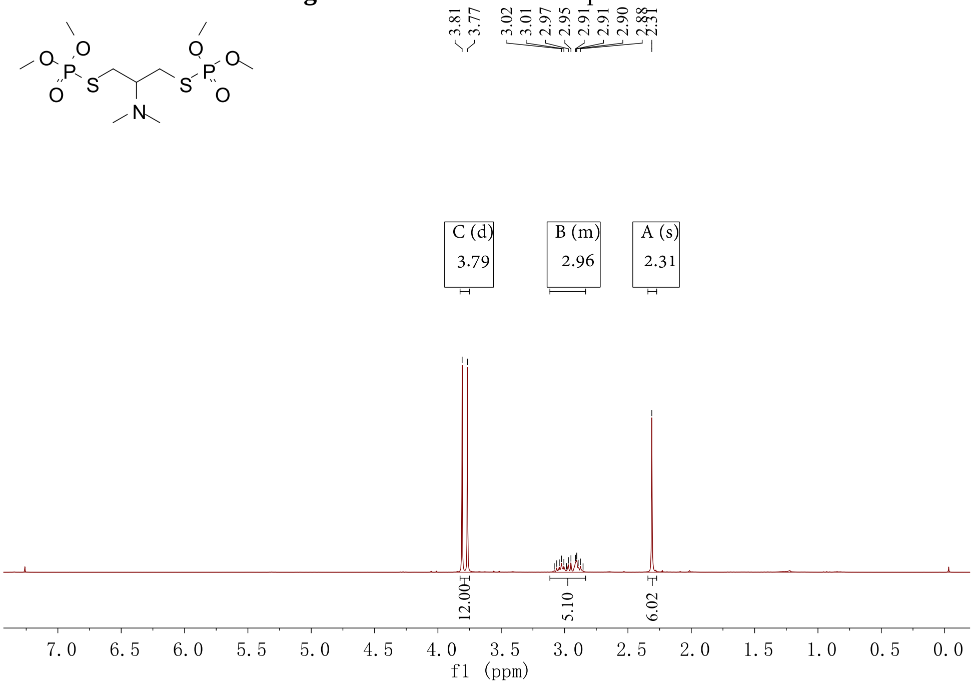

Figure S2. ^{1}H-NMR of compound **7a**.

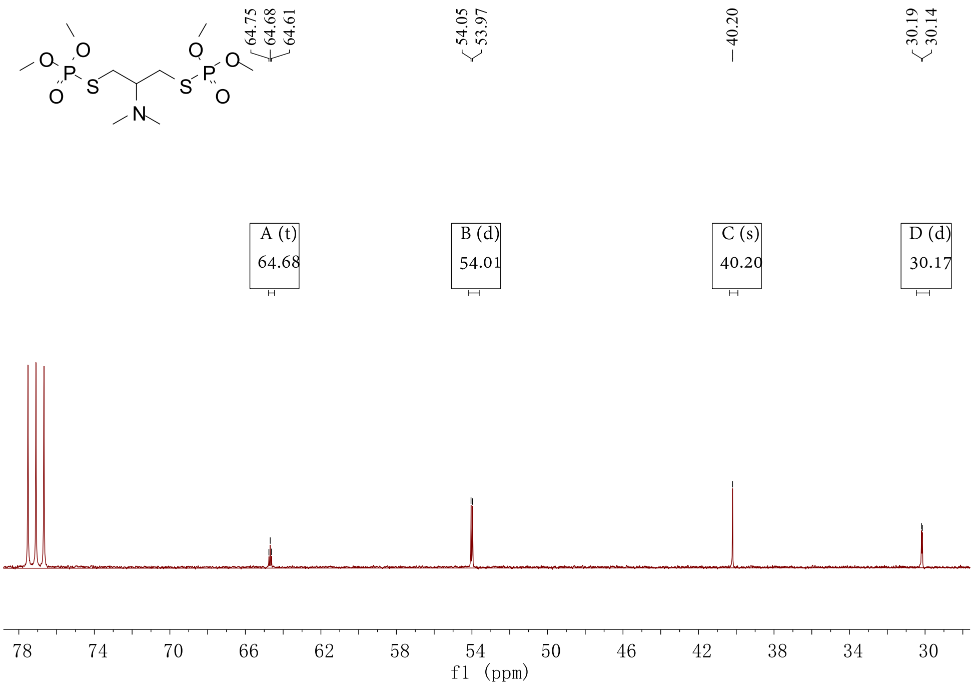

Figure S3. ^{13}C-NMR of compound **7a**.

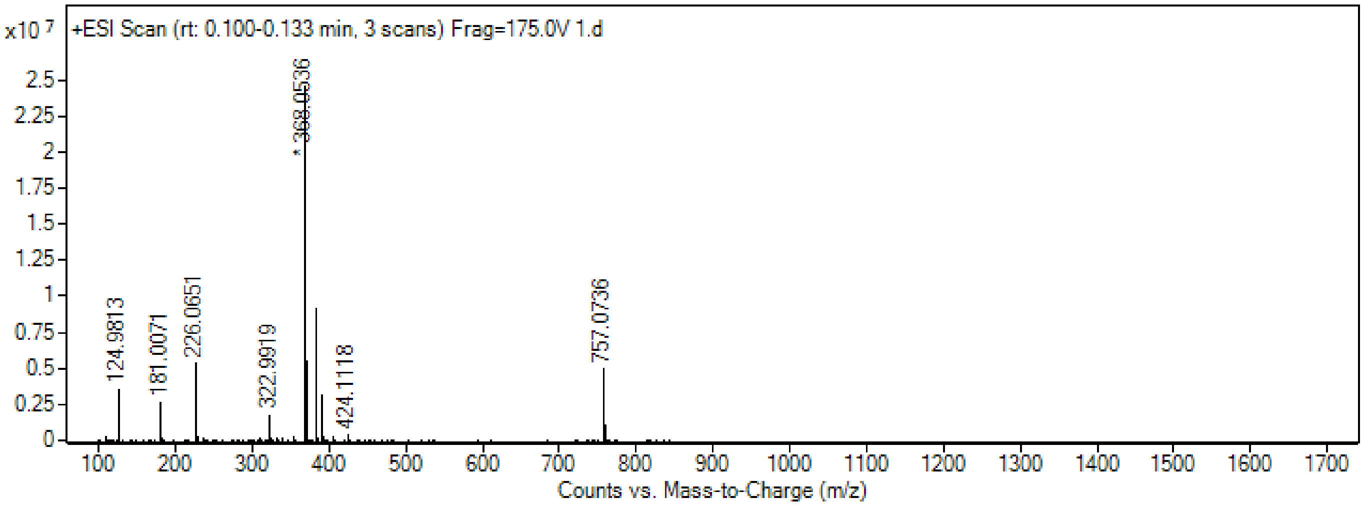

Figure S4. HRMS of compound **7a**.

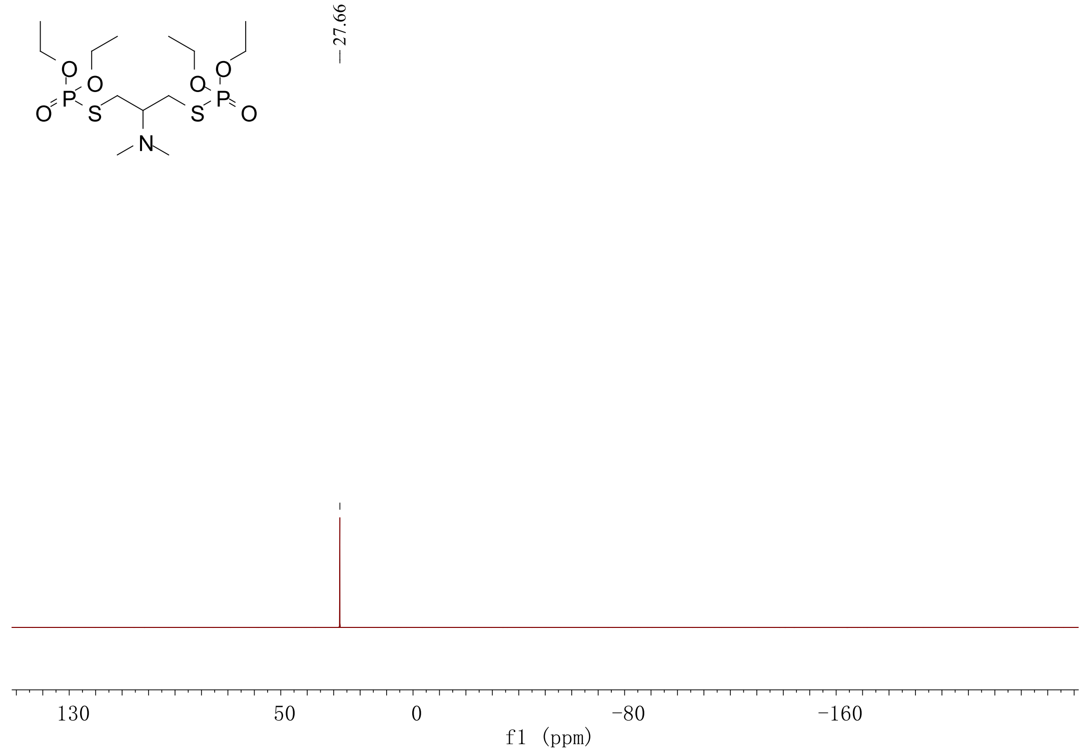

Figure S5. ^{31}P-NMR of compound **7b**.

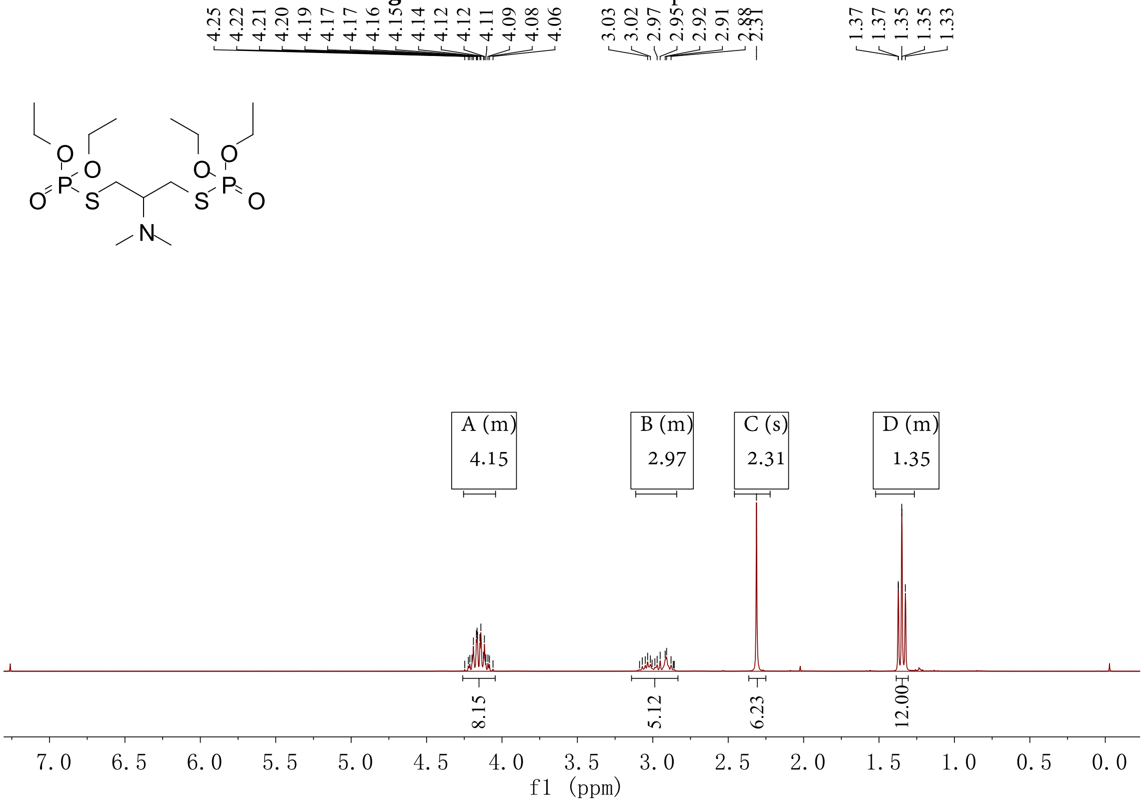

Figure S6. ^{1}H-NMR of compound **7b**.

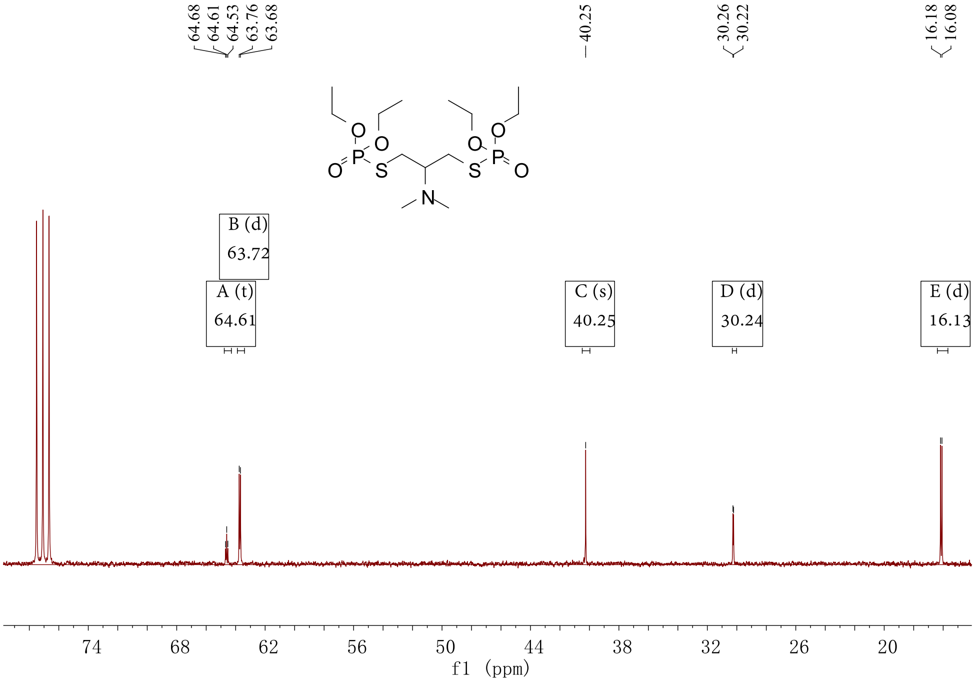

Figure S7. ^{13}C-NMR of compound **7b**.

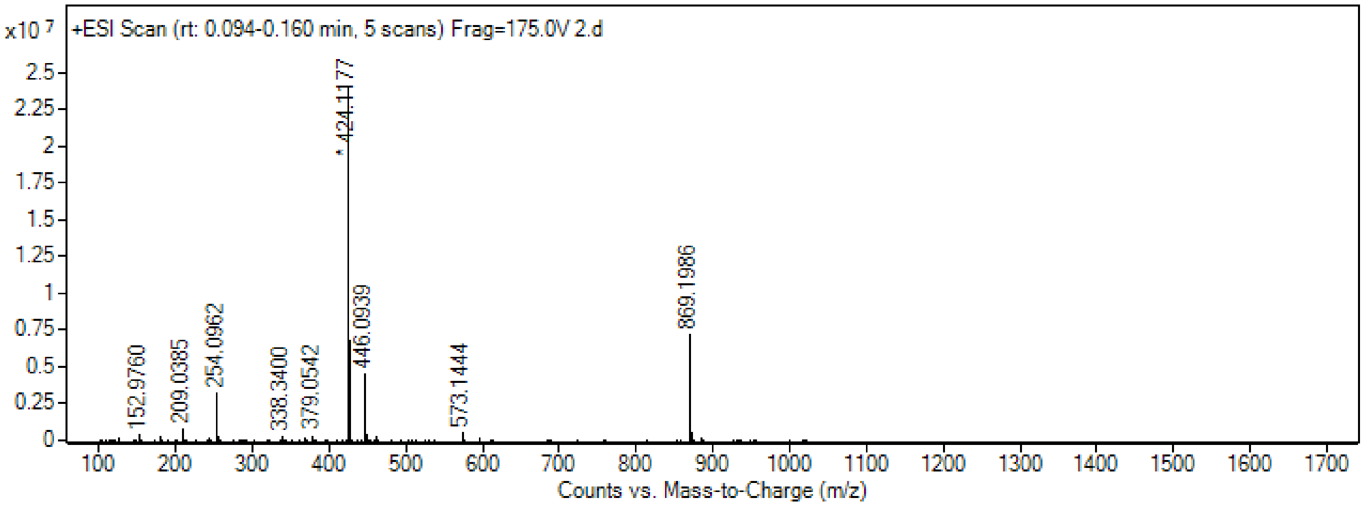

Figure S8. HRMS of compound **7b**.

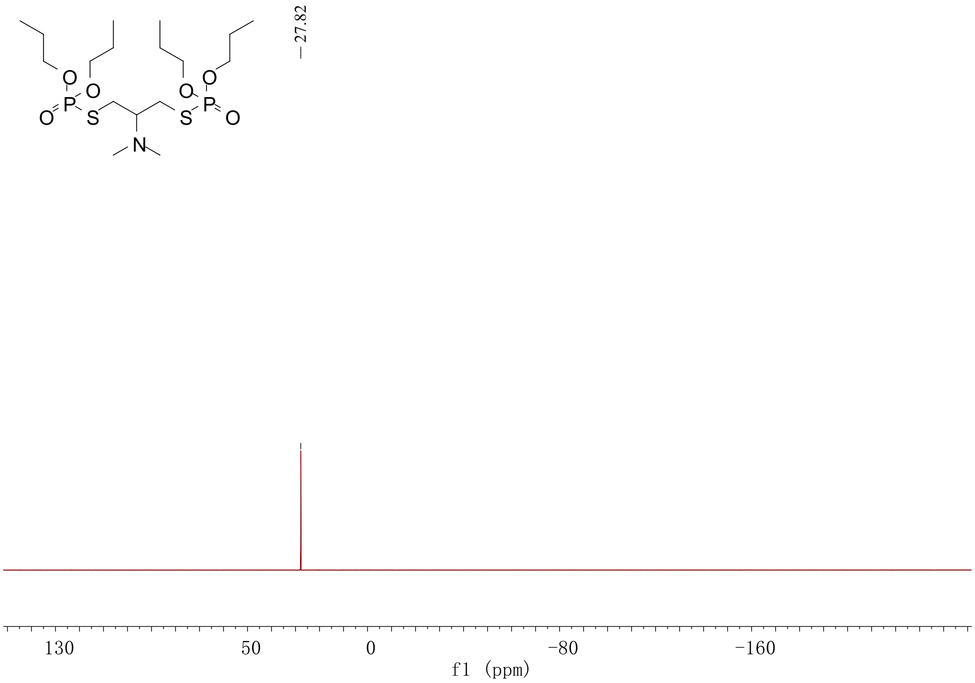

Figure S9. ^{31}P-NMR of compound **7c**.

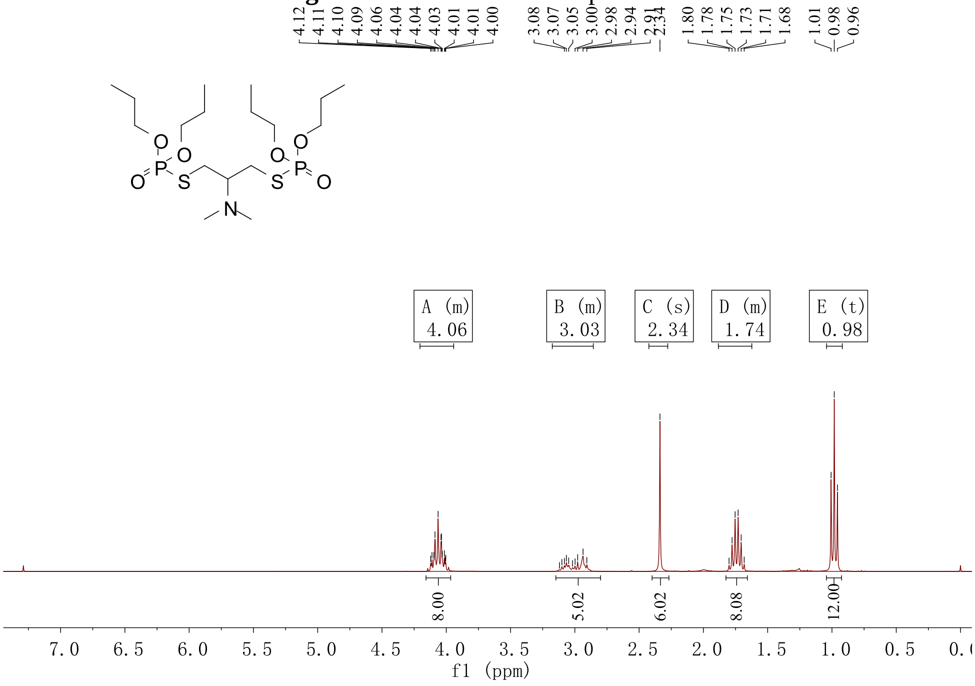

Figure S10. ^{1}H-NMR of compound **7c**.

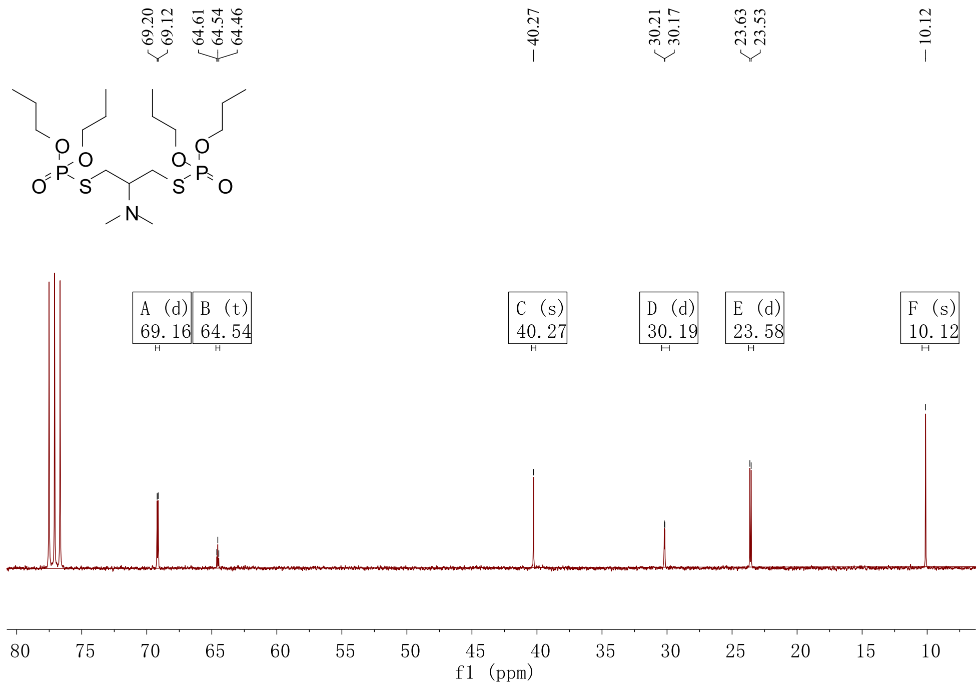

Figure S11. ^{13}C-NMR of compound **7c**.

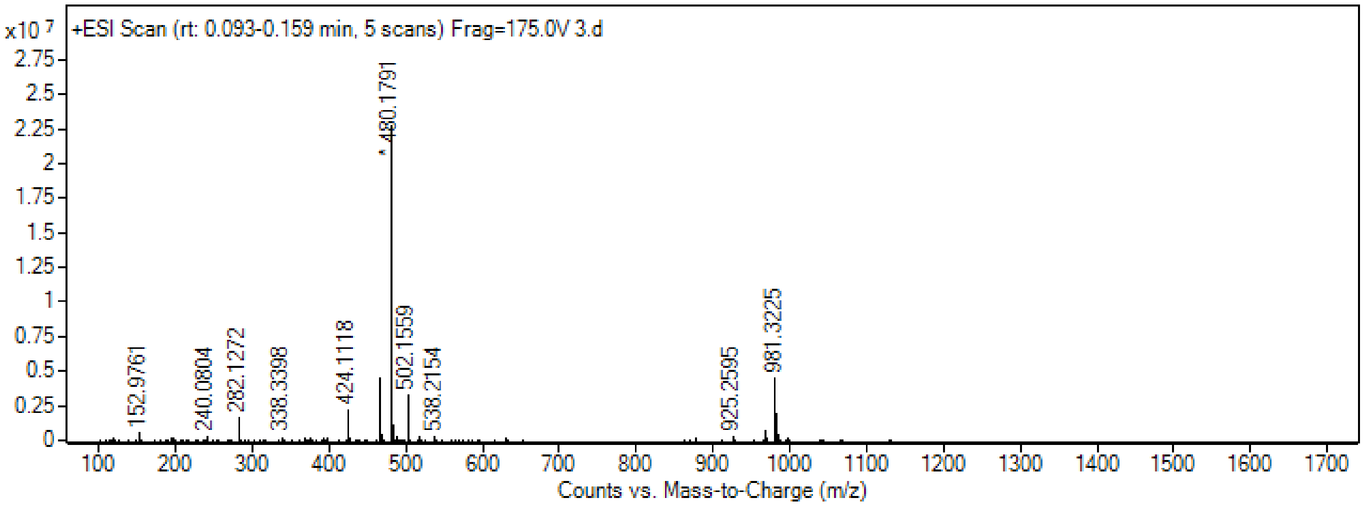

Figure S12. HRMS of compound **7c**.

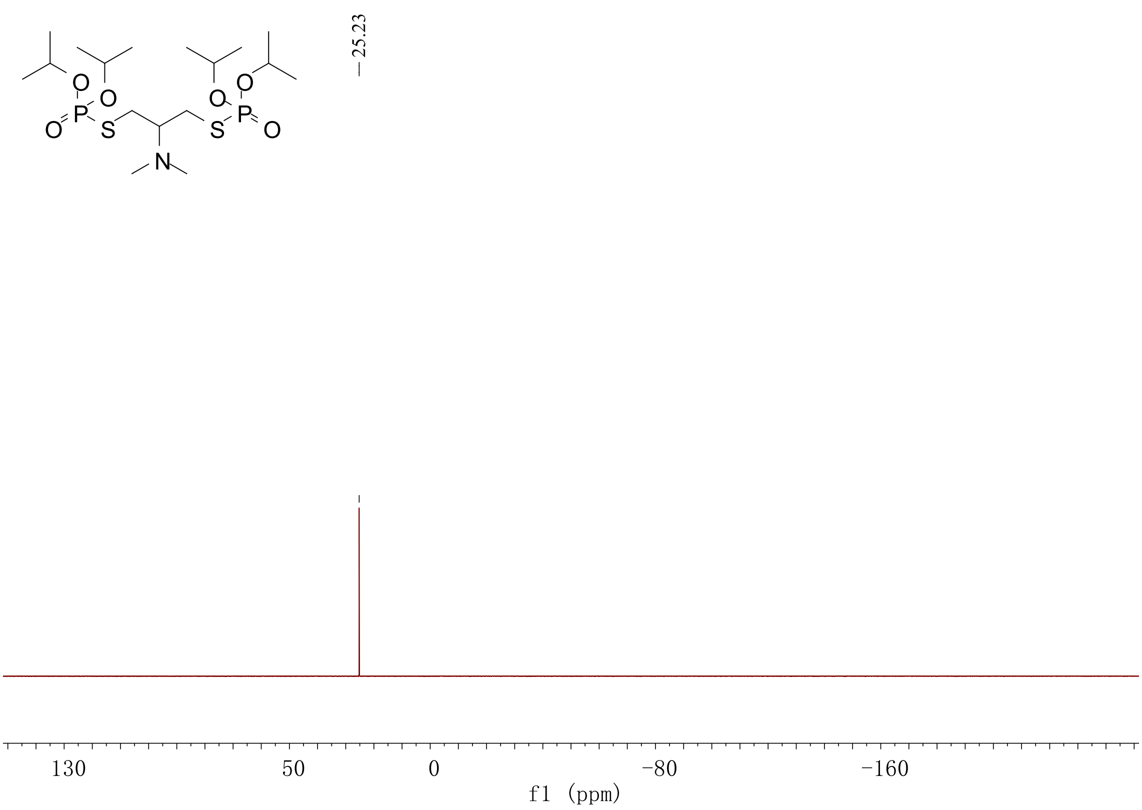

Figure S13. ^{31}P-NMR of compound **7d**.

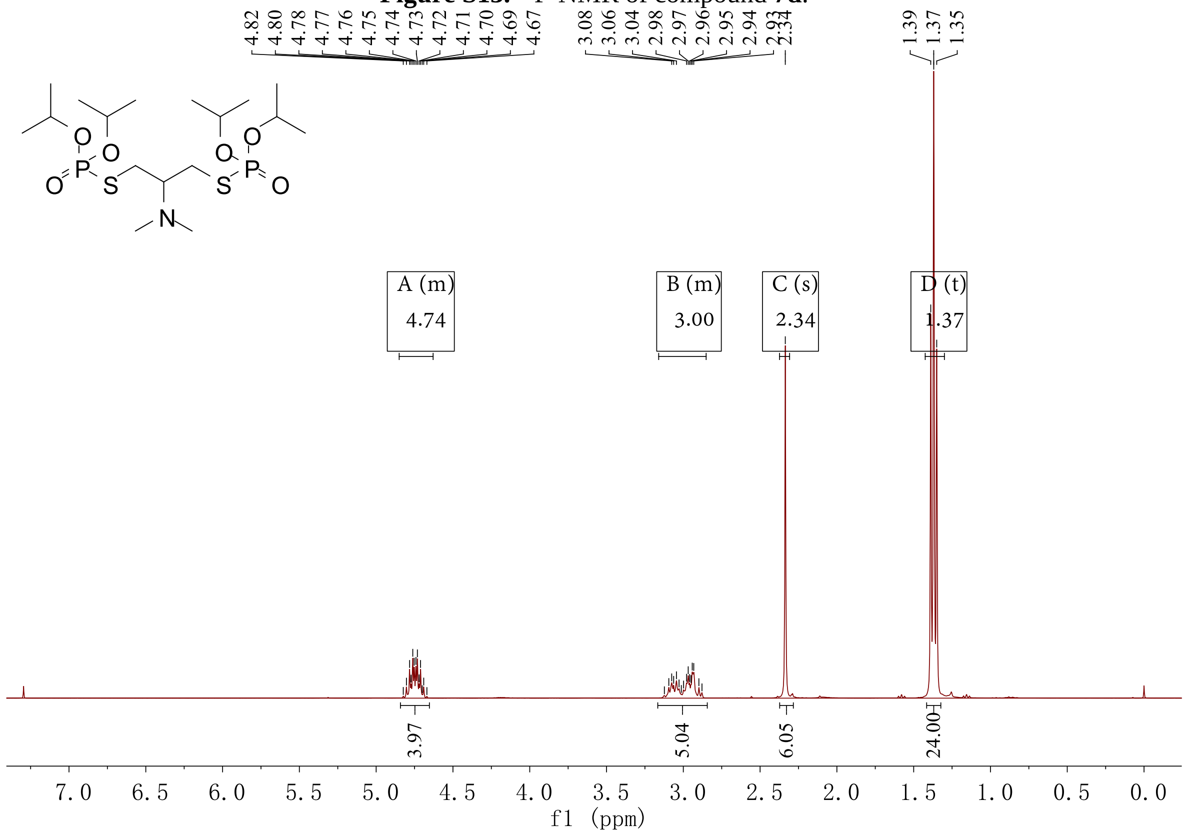

Figure S14. ^{1}H-NMR of compound **7d**.

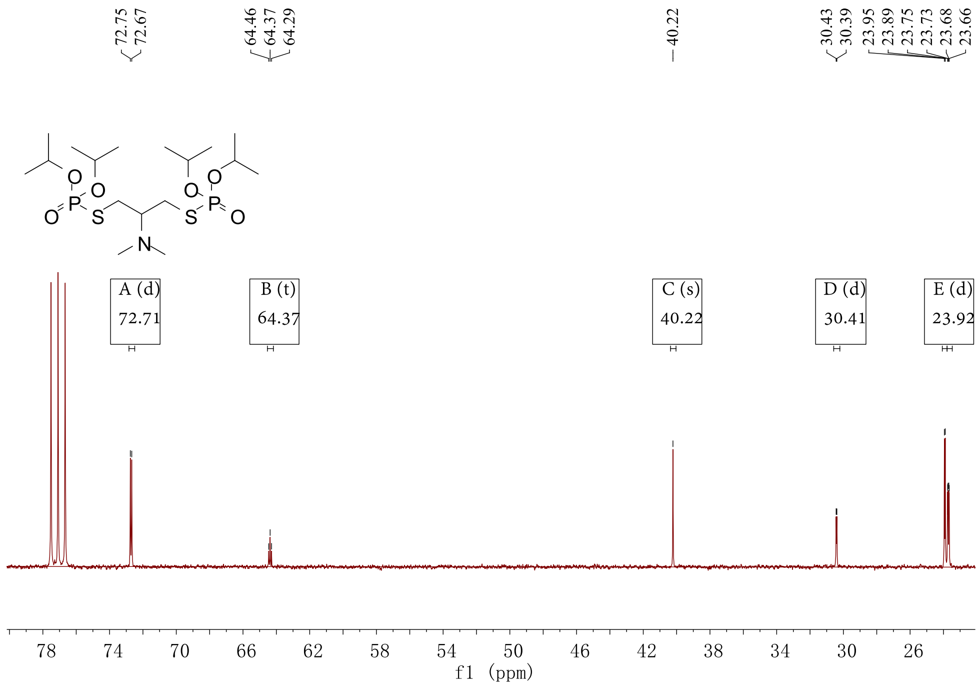

Figure S15. ^{13}C-NMR of compound **7d**.

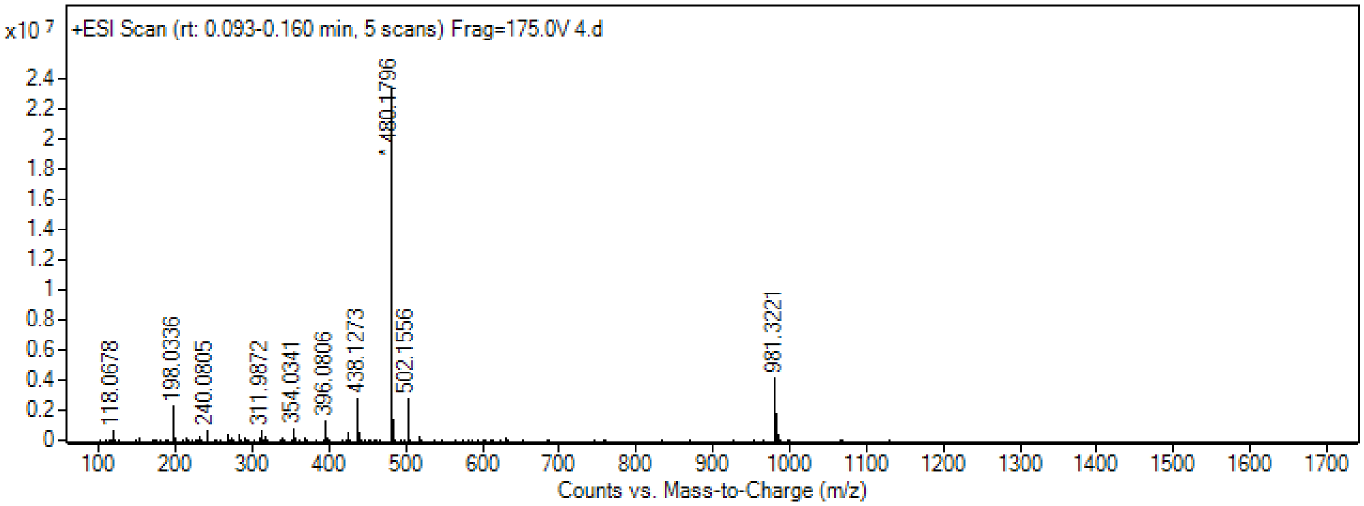

Figure S16. HRMS of compound **7d**.

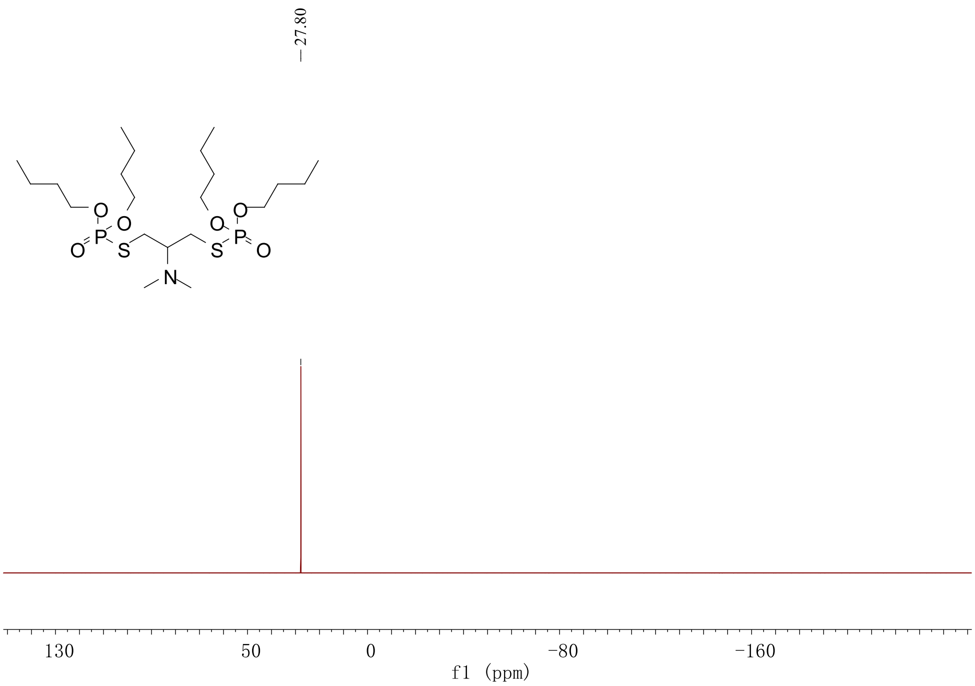

Figure S17. ^{31}P-NMR of compound **7e**.

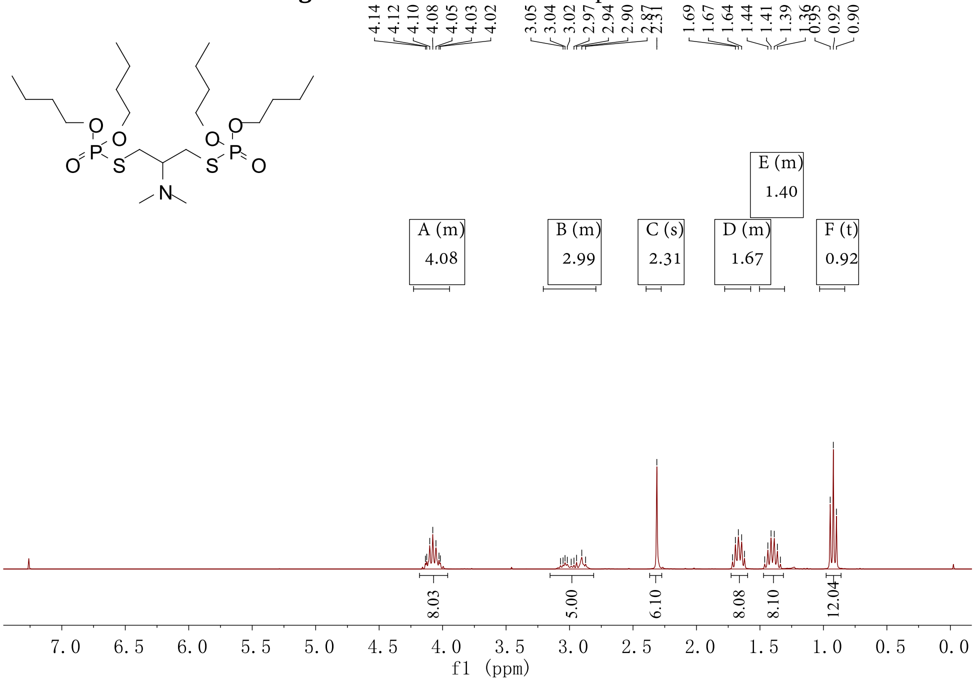

Figure S18. ^{1}H-NMR of compound **7e**.

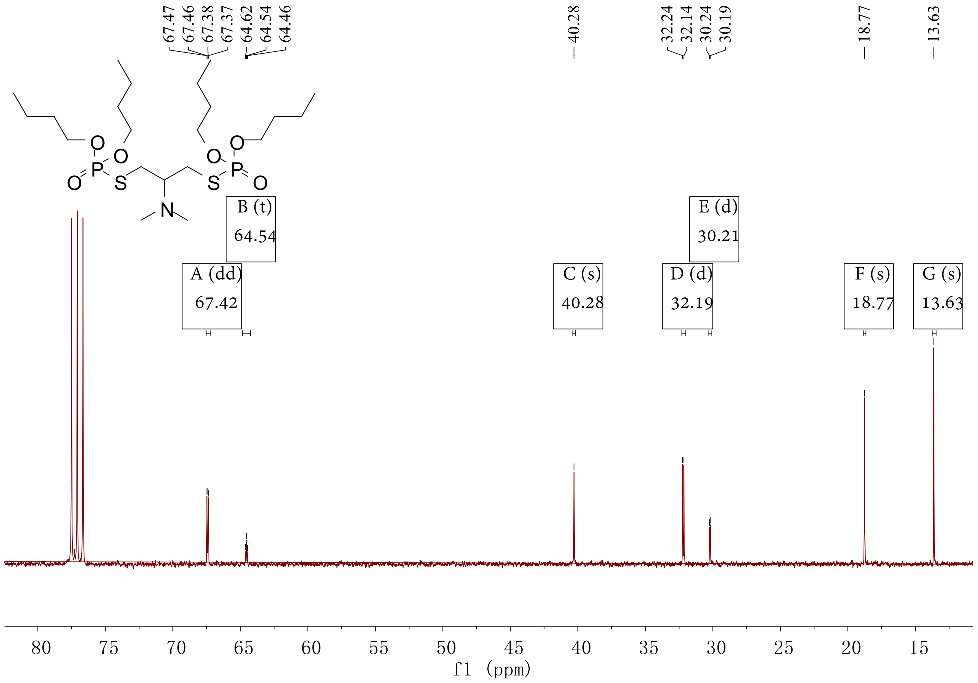

Figure S19. ^{13}C-NMR of compound **7e**.

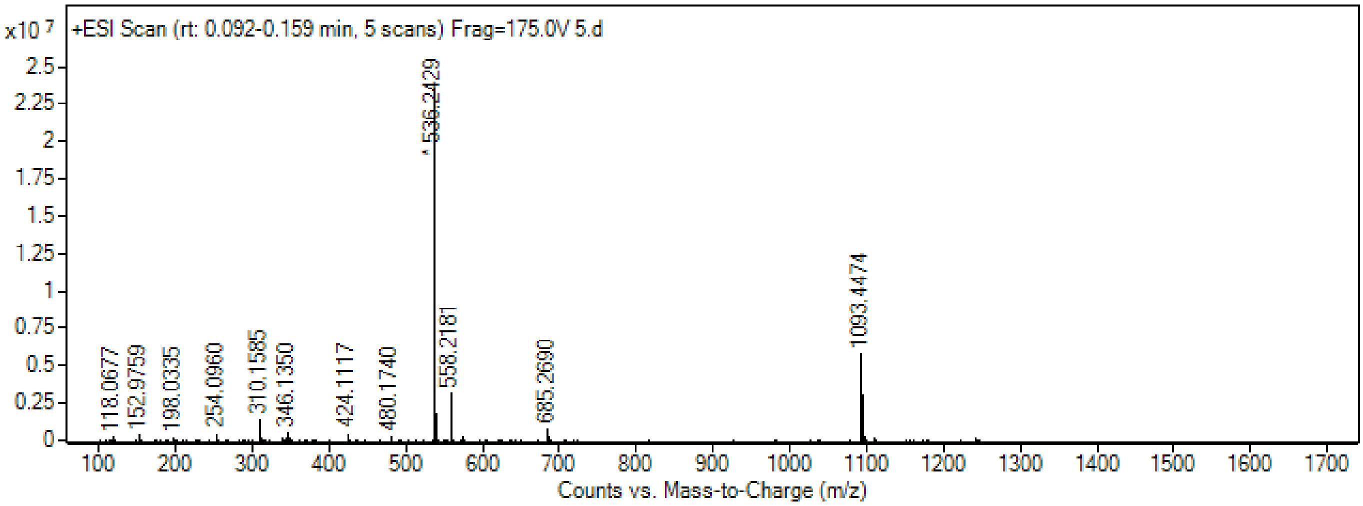

Figure S20. HRMS of compound **7e**.

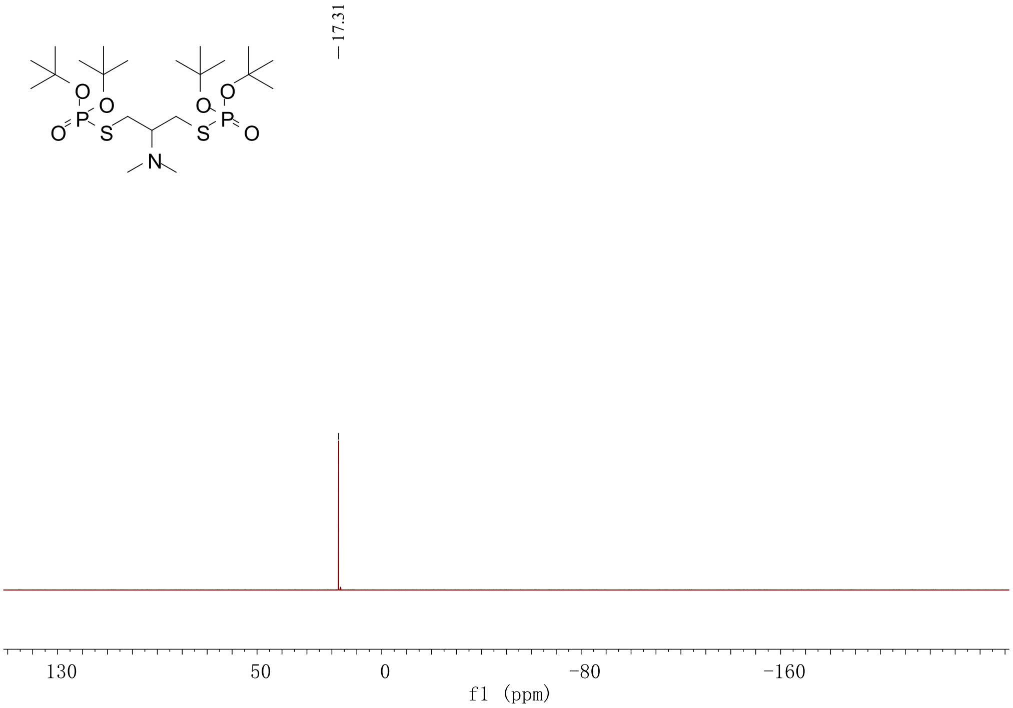

Figure S21. ^{31}P-NMR of compound **7f**.

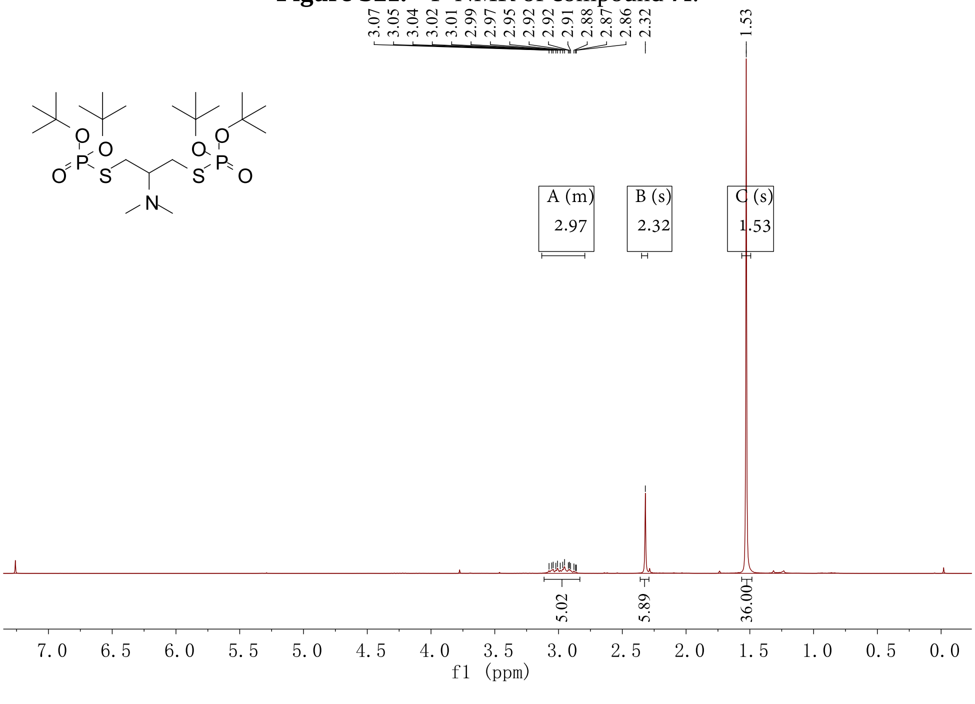

Figure S22. ^{1}H-NMR of compound **7f**.

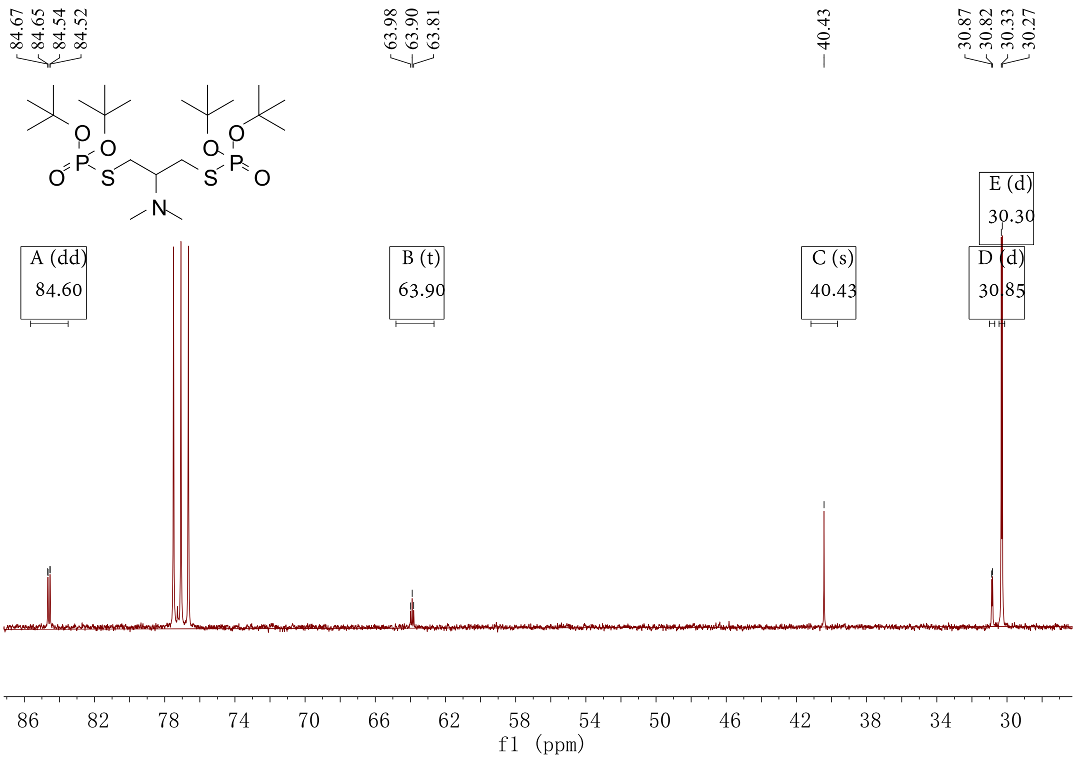

Figure S23. ^{13}C-NMR of compound **7f**.

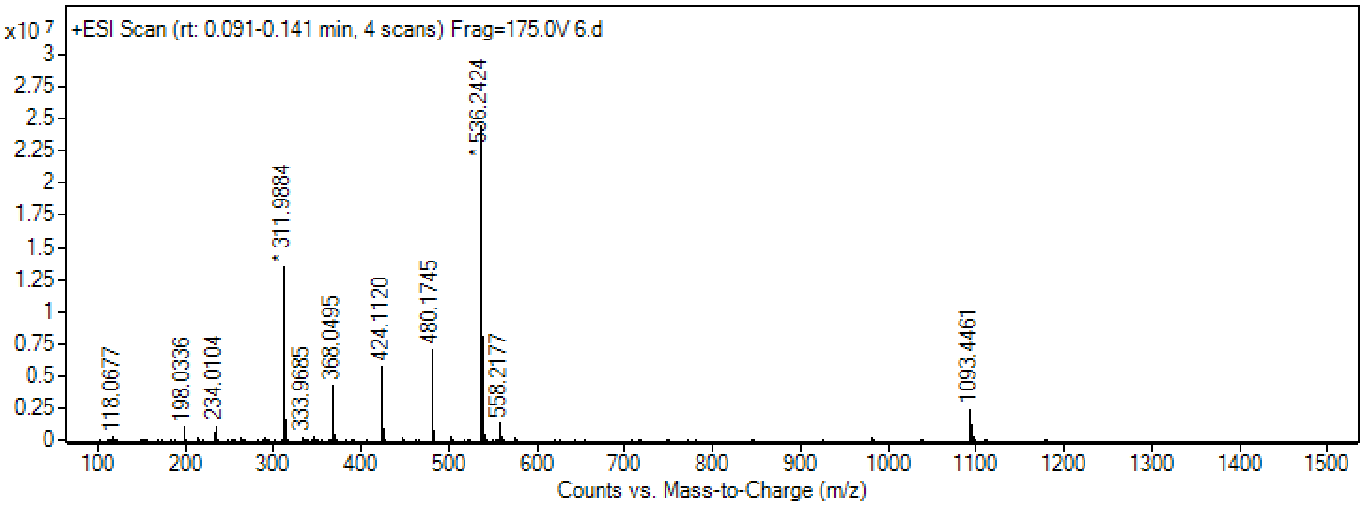

Figure S24. HRMS of compound **7f**.

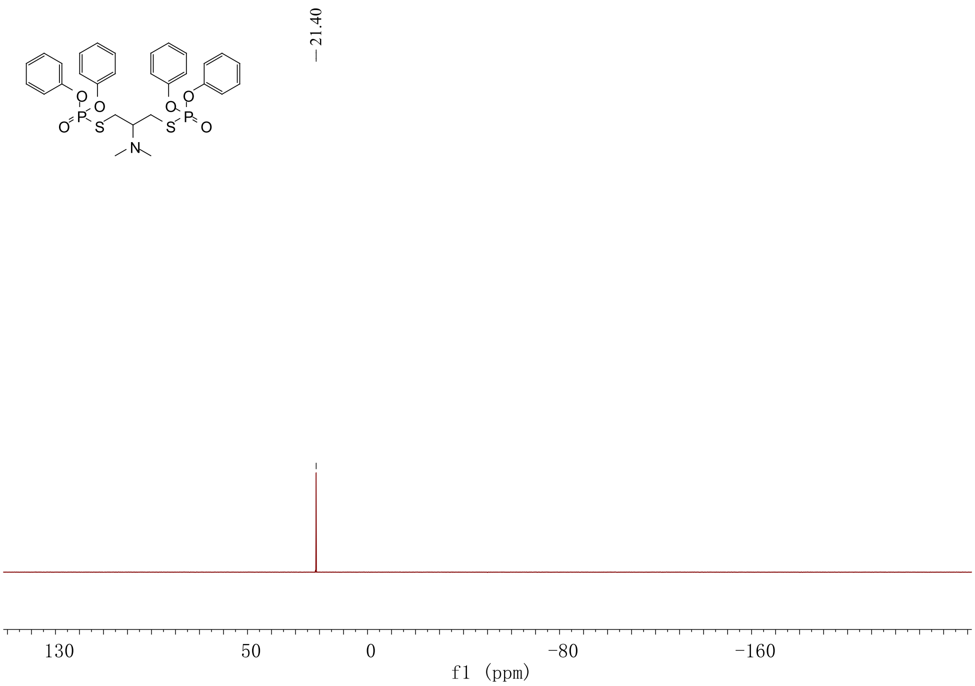

Figure S25. ^{31}P-NMR of compound **7g**.

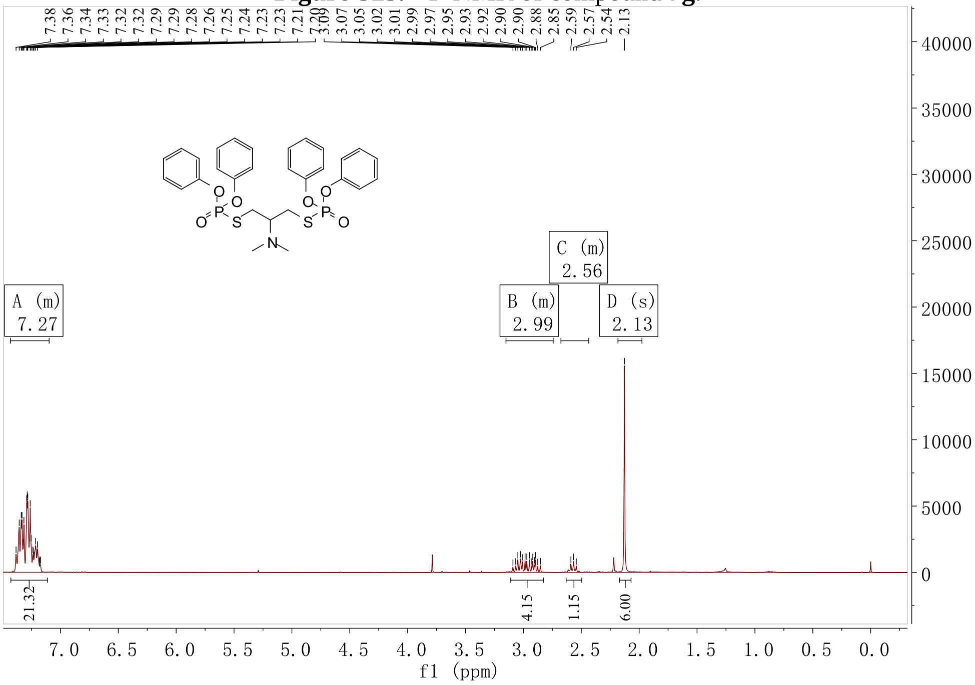

Figure S26. ^{1}H-NMR of compound **7g**.

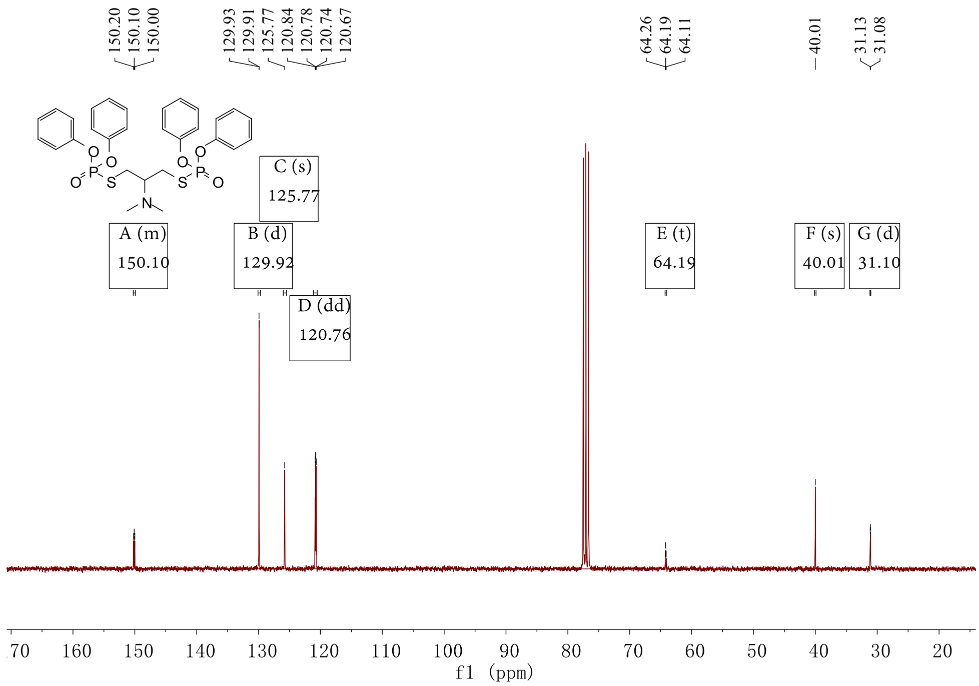

Figure S27. ^{13}C-NMR of compound **7g**.

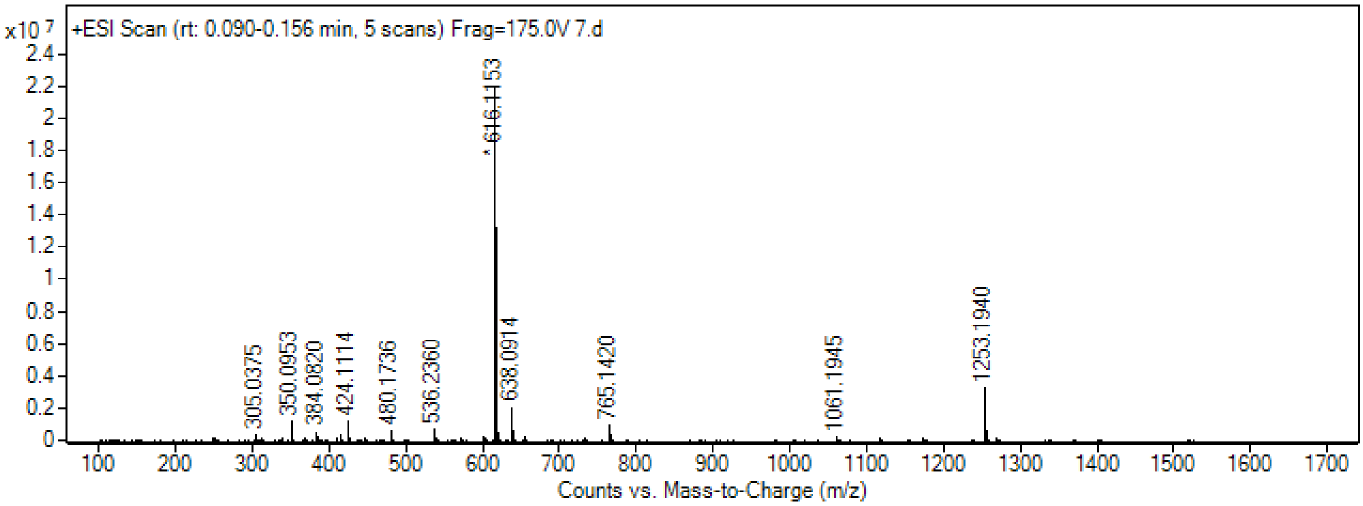

Figure S28. HRMS of compound **7g**.

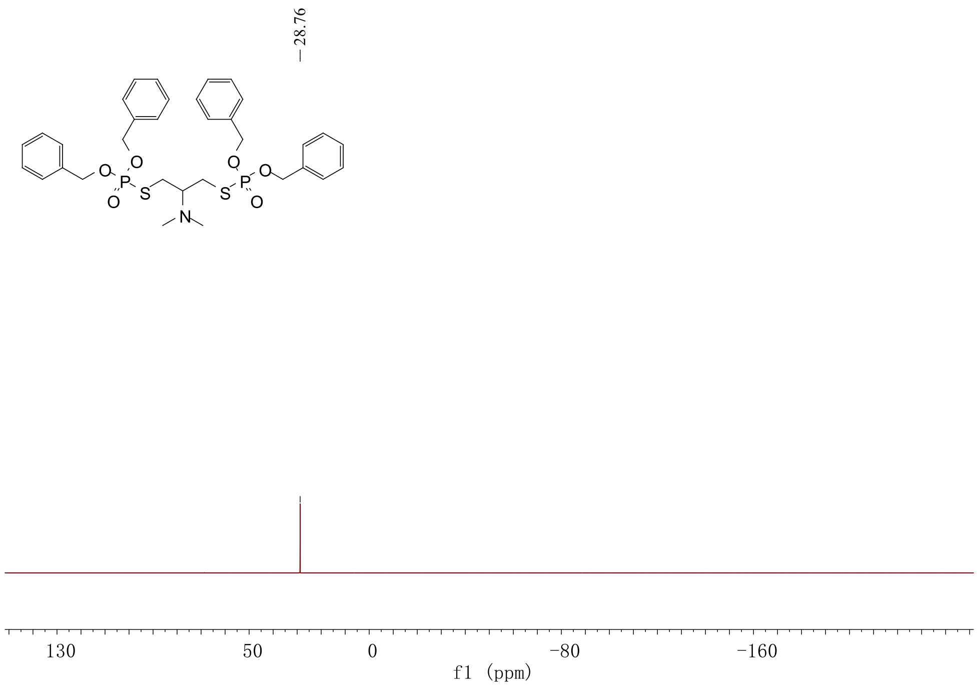

Figure S29. ^{31}P-NMR of compound **7h**.

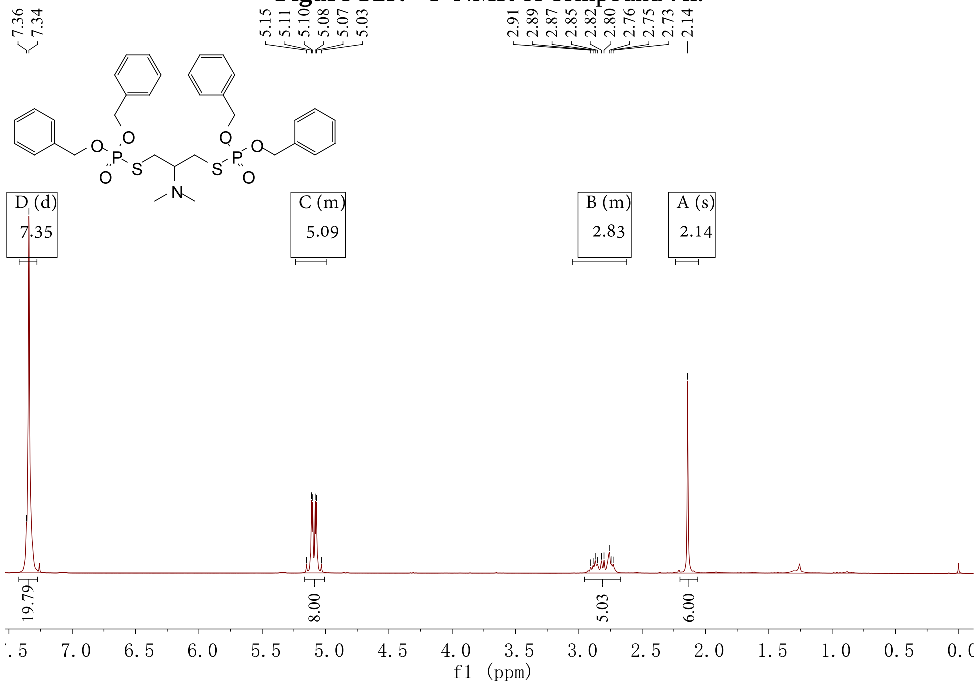

Figure S30. ^{1}H-NMR of compound **7h**.

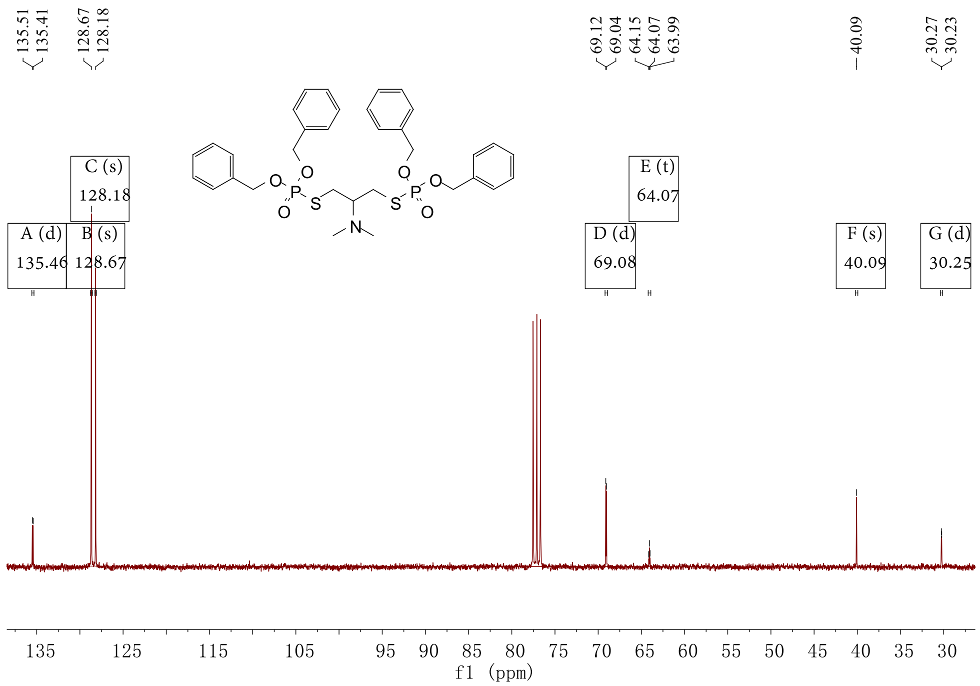

Figure S31. ^{13}C-NMR of compound **7h**.

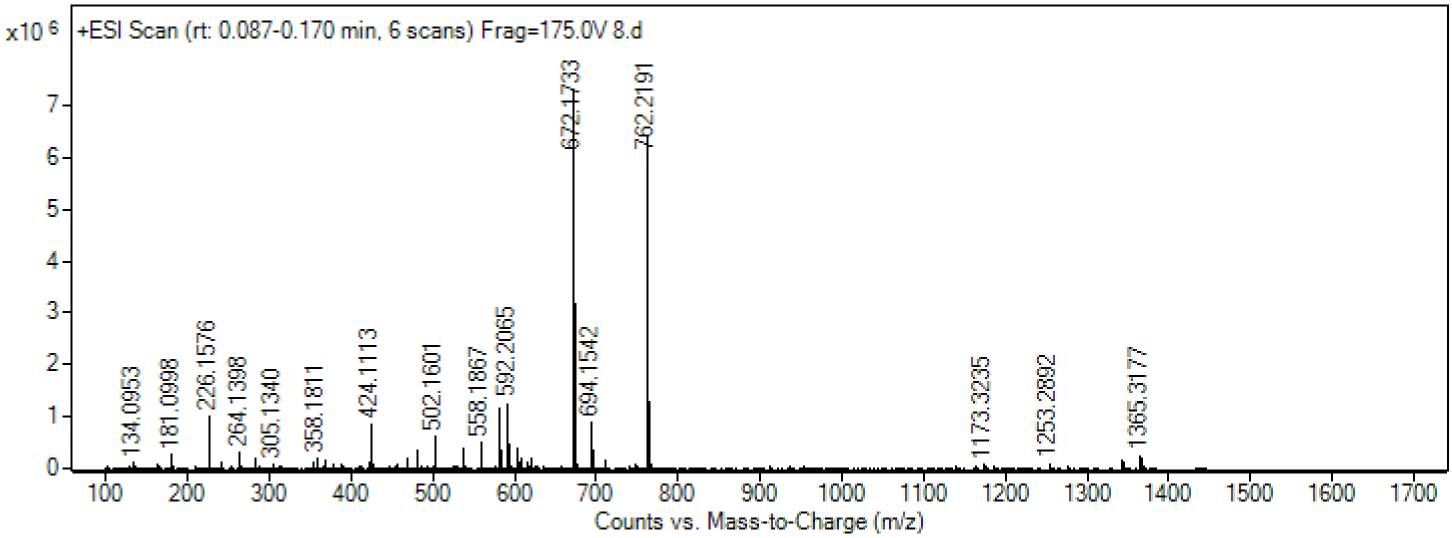

Figure S32. HRMS of compound **7h**.

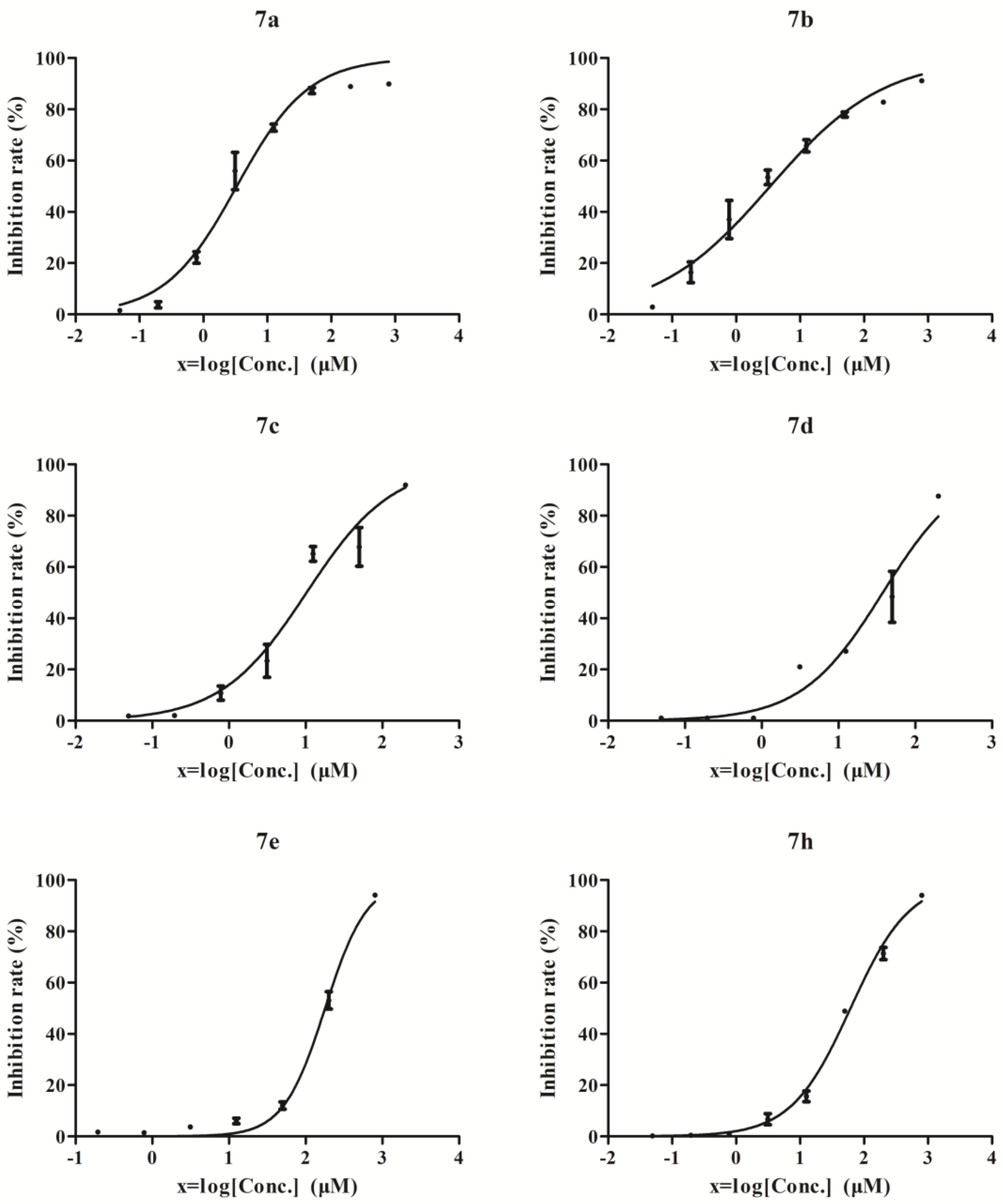

Figure S33. The concentration-inhibition rate relationship of **7a**, **7b**, **7c**, **7d**, **7e**, **7h**.

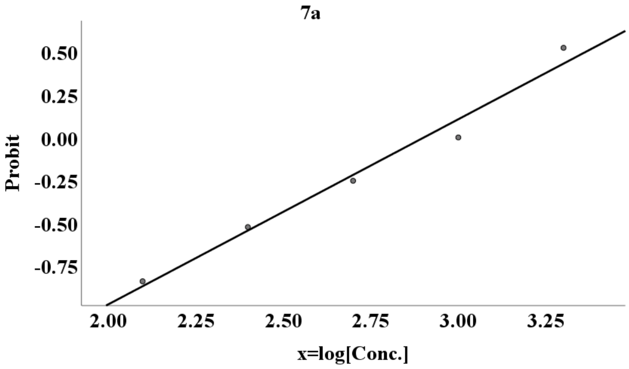

Figure S34. The concentration-corrected mortality rate (against M. separata) relationship of **7a**, **7b**, **7c**, **7f**, **7g**, nereistoxin and chlorpyrifos.

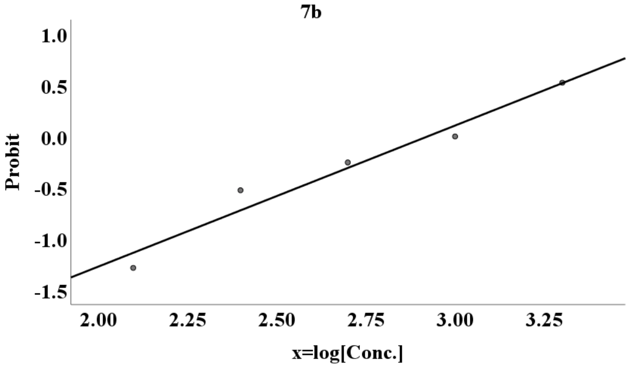

Figure S35. The concentration-corrected mortality rate (against M. separata) relationship of **7a**, **7b**, **7c**, **7f**, **7g**, nereistoxin and chlorpyrifos.

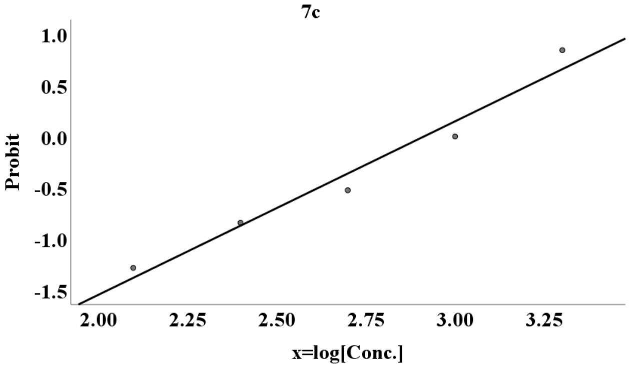

Figure S36. The concentration-corrected mortality rate (against M. separata) relationship of **7a**, **7b**, **7c**, **7f**, **7g**, nereistoxin and chlorpyrifos.

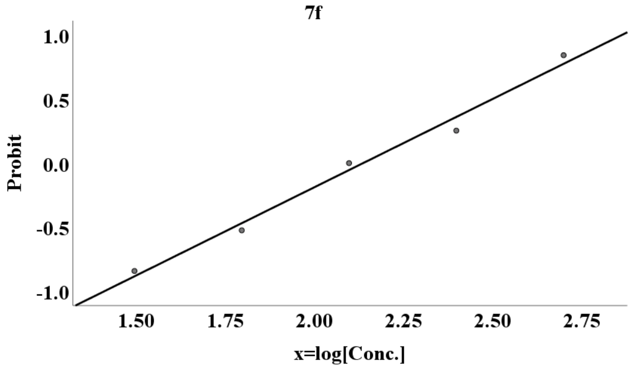

Figure S37. The concentration-corrected mortality rate (against M. separata) relationship of **7a**, **7b**, **7c**, **7f**, **7g**, nereistoxin and chlorpyrifos.

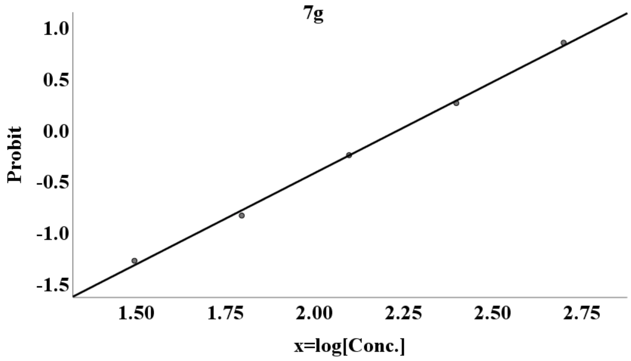

Figure S38. The concentration-corrected mortality rate (against M. separata) relationship of **7a**, **7b**, **7c**, **7f**, **7g**, nereistoxin and chlorpyrifos.

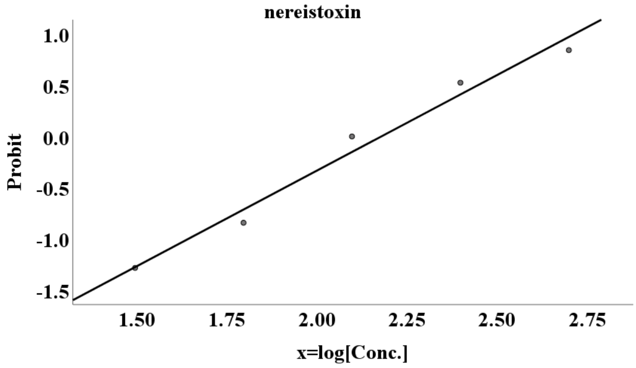

Figure S39. The concentration-corrected mortality rate (against M. separata) relationship of **7a**, **7b**, **7c**, **7f**, **7g**, nereistoxin and chlorpyrifos.

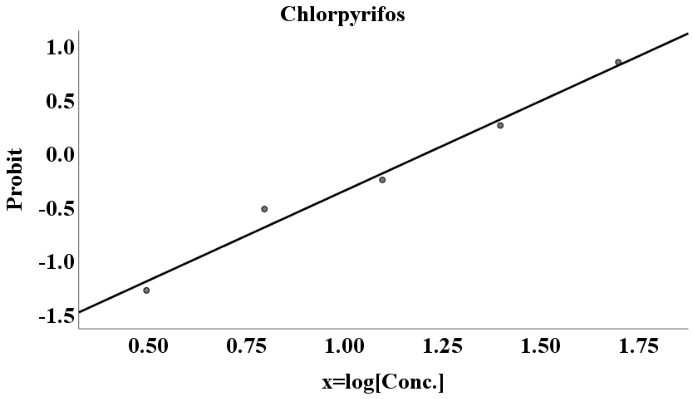

Figure S40. The concentration-corrected mortality rate (against M. separata) relationship of **7a**, **7b**, **7c**, **7f**, **7g**, nereistoxin and chlorpyrifos.

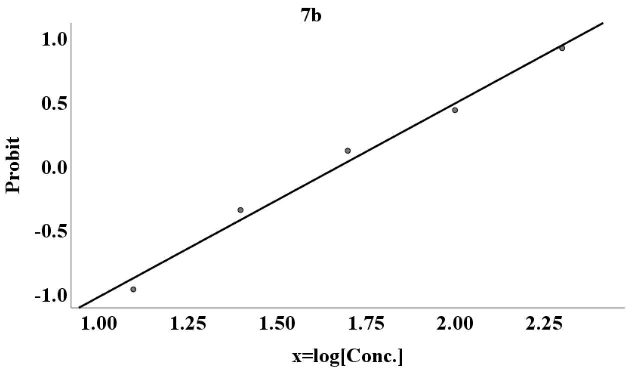

Figure S41. The concentration-corrected mortality rate (against M. persicae) relationship of **7b**, **7f** and thiamethoxam.

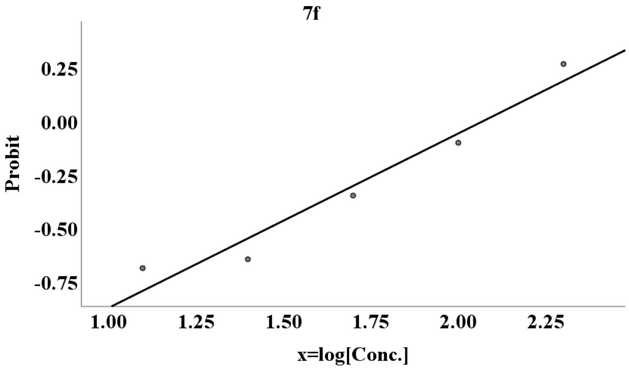

Figure S42. The concentration-corrected mortality rate (against M. persicae) relationship of **7b**, **7f** and thiamethoxam.

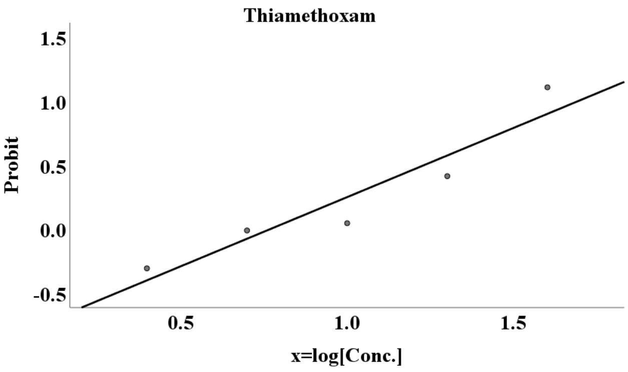

Figure S43. The concentration-corrected mortality rate (against M. persicae) relationship of **7b**, **7f** and thiamethoxam.

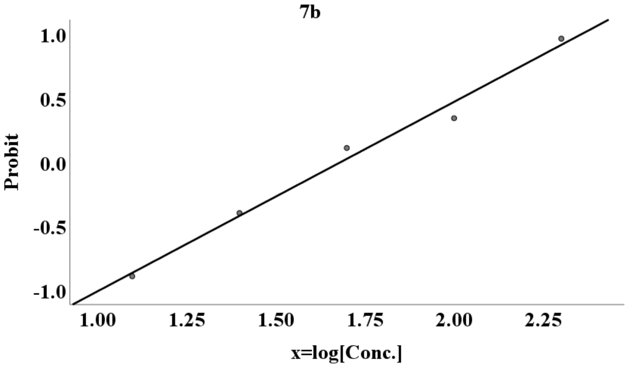

Figure S44. The concentration-corrected mortality rate (against M. persicae) relationship of **7b**, **7f** and thiamethoxam.

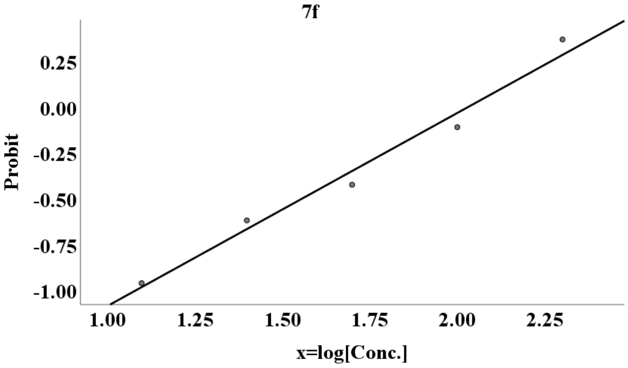

Figure S45. The concentration-corrected mortality rate (against M. persicae) relationship of **7b**, **7f** and thiamethoxam.

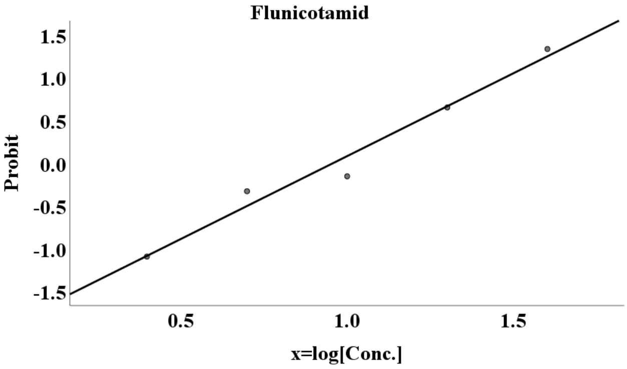

Figure S46. The concentration-corrected mortality rate (against R. padi) relationship of **7b**, **7f** and flunicotamid.
